# Comparison of Color Development Kinetics of Tanning Reactions of Dihydroxyacetone with Free and Protected Basic Amino Acids

**DOI:** 10.1021/acsomega.2c06124

**Published:** 2022-12-01

**Authors:** Yufa Sun, Subin Lee, Long Lin

**Affiliations:** Colour Science, School of Chemistry, University of Leeds, Woodhouse Lane, LeedsLS2 9JT, U.K.

## Abstract

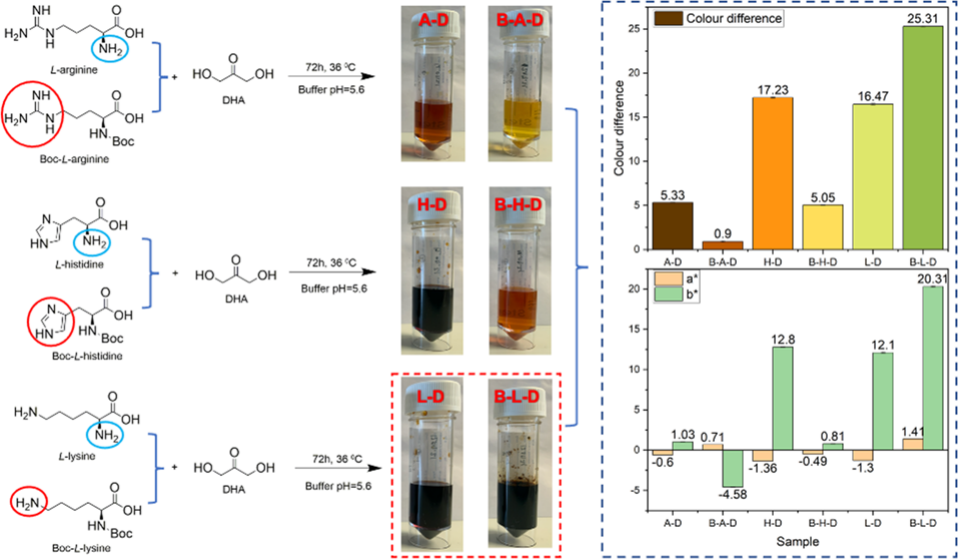

Sunless tanning has become incredibly prevalent due to the increasing fashionable demand and the awareness of photodamage risks. The brown pigments are induced by dihydroxyacetone (DHA) and amino groups in the stratum corneum (SC) of skin via the Maillard reaction. While most studies concerning sunless tanning reactions have focused on free amino acids (AAs), little information is available on the impact of the side chain of AAs or proteins on this important reaction in cosmetic chemistry. To explore the reactivity and color development kinetics of different types of amino groups, three basic free AAs (Arg, His, and Lys) and three Nα-protected AAs (Boc-Arg-OH, Boc-His-OH, and Boc-Lys-OH) were used to react with DHA using a simplified model system at different reaction times, pH, and temperatures. Full factorial experiments were employed to design and analyze the effects of these three factors. The browning intensity and color characteristics were quantitatively evaluated. The factorial experiments showed that temperature had the most significant influence on the browning intensity and played a dominant role in the interactions with the reaction time and pH. It was found, for the first time, that Arg and His reacted with DHA more rapidly than Boc-Arg-OH and Boc-His-OH, while Boc-Lys-OH developed a stronger color than Lys under the same conditions, suggesting that ε-NH_2_ of a lysine residue in peptides or proteins of SC may play a crucial role in the color development of DHA tanning. This study not only clearly illustrates the capability of the side chain of AAs to produce colored compounds but also provides a deeper understanding of DHA tanning.

## 1 Introduction

The public interest in tanning has grown dramatically since more and more people view the tanned skin as more esthetically pleasing and healthy.^1,2^ Various ways of tanning have emerged and evolved through the years to become more readily feasible and convenient to use for all consumers, such as natural ultraviolet radiation (UVR), artificial UVR, and sunless tanning products.^3^ With the increasing incidence of skin cancer, people’s desire for safer and more efficient tanning methods has made sunless tanning the most popular way.^4,5^ Sunless tanning products not only produce a durable sun-kissed look without the risks of photodamage but also offer a moderate sun protection factor.^6,7^ These products come in many forms, such as lotions, mousses, gels, and creams.

Dihydroxyacetone (DHA), the simplest ketose, is the main active ingredient, which is derived from plants and commercially obtained by the microbial fermentation of glycerol.^8,9^ The chemistry of DHA tanning is similar to the well-known Maillard reaction in food.^10,11^ The first person to draw a connection between the browning in foods and the DHA tanning on skin is Dr Eva Wittgenstein, by accident, while using DHA as an oral drug to assist children with glycogen storage disease.^12,13^ It has been widely believed that DHA reacts chemically with free amino acids (AAs) in the stratum corneum (SC) via the Maillard reaction to produce brown pigments, also called melanoidins.^14^ DHA has been recognized by the Food and Drug Administration (FDA) of US and EU Scientific Committee on Consumer Safety as a safe skin coloring agent in cosmetic and even been proven to be helpful in the treatment of vitiligo.^15−17^

Three basic AAs, arginine (Arg), histidine (His), and lysine (Lys), have been reported to be abundant in the epidermal proteins of SC and have a high reactivity with DHA.^18^ In our previous study, the color development of the Maillard reaction between these AAs and DHA has been investigated under various reaction conditions.^19^ However, many recent studies have drawn our attention and suggested that the side chain of α-keratin in SC plays a more important role in this reaction, since the formed color is resistant to normal water, soap, and sweat exposure, and it even lasts for 5–7 days.^2,20^ Even from free AAs, the final color bodies should involve high molecular weight species, which will be substantive in skin. In this case, the reaction site of DHA on α-NH_2_ of free AAs becomes less important and the nucleophilic side chains of AAs consisting of peptide or protein may play a dominant role, particularly the ε-amino group of lysine and the guanidino side chain of arginine.^21,22^ In addition, the Maillard reactions between sugars (such as glucose and fructose) and arginine- and lysine-containing peptides or proteins in food and the human body have been intensively investigated, but the related reactions between DHA and the peptides or proteins are rarely reported.^23−26^

Considering the complex structures and synthesis difficulties of peptides or proteins, three AAs with α-NH_2_ protected with tert-butyloxycarbonyl (Boc-AAs), Boc-Arg-OH, Boc-His-OH, and Boc-Lys-OH, were chosen to simplify the reaction route and better explore the color development of DHA with the side chain of AAs. Meanwhile, this study also investigated the differences in color development between AAs and Boc-AAs under different reaction conditions to predict and comprehend inflectional factors and possible reaction routes of DHA tanning reactions. The color characteristics and color differences of these model systems were quantitatively studied based on the change of their CIE L*a*b* values as the reaction progressed under various conditions. Minitab was used to design factorial experiments and analyze the experimental data obtained to study factors that have significant effects on the color development kinetics. To the best of our knowledge, this is the first time to systematically study the color formation of DHA with the side chain of AAs using a simplified model, which is of great significance for a deeper understanding and interpretation of DHA tanning on human skin.

## 2 Experimental Section

### 2.1 Chemicals

**Figure 1:**
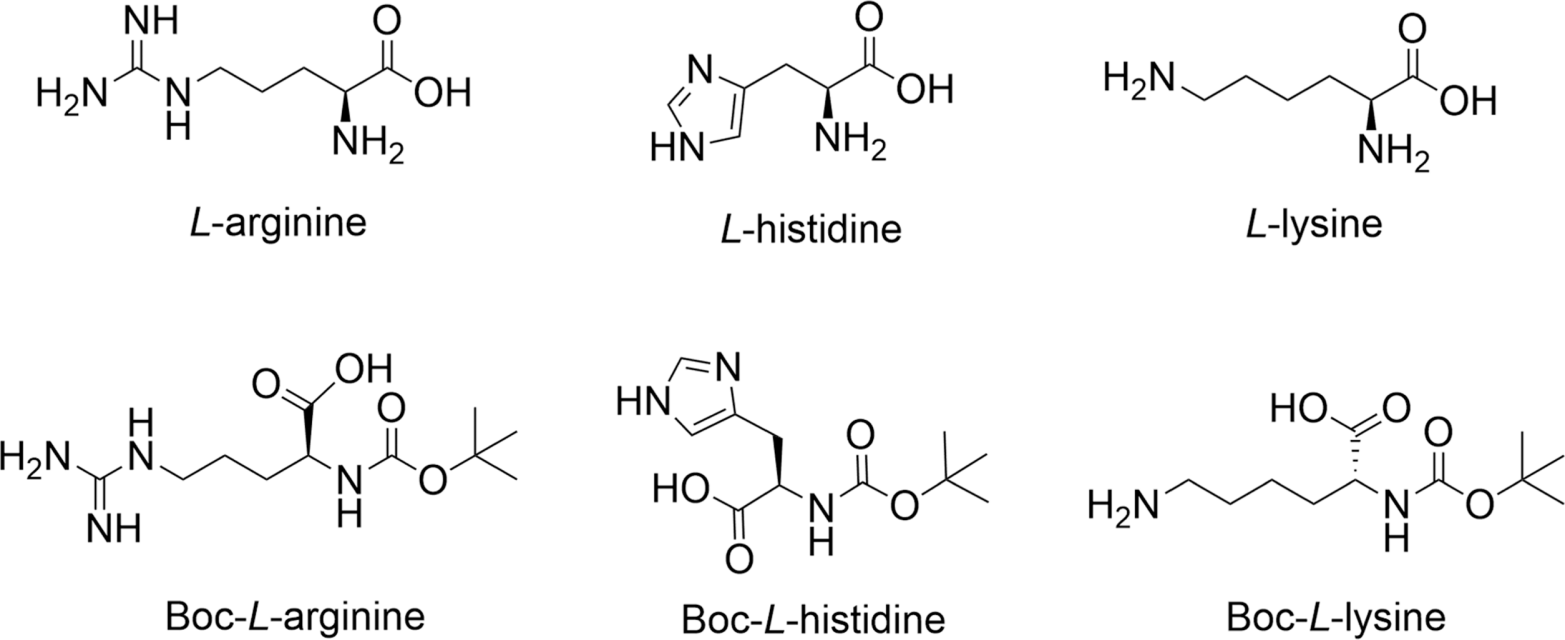
Chemical structures of studied three basic amino acids and their α-amino group-protected derivatives.

Dihydroxyacetone (DHA) solution was supplied by PZCussons (Manchester, England). l-Arginine hydrochloride (Arg), l-histidine hydrochloride (His), and l-lysine hydrochloride (Lys) were purchased from Ajinomoto Inc. *N*-(*tert*-Butoxycarbonyl)-l-arginine (Boc-Arg-OH), *N*-(*tert*-butoxycarbonyl)-l-histidine (Boc-His-OH), and *N*-(*tert*-butoxycarbonyl)-l-lysine (Boc-Lys-OH) were analytical grade and purchased from Fluorochem Limited. Their chemical structures are summarized in Figure 1. Hydrochloric acid (HC1, 36.5%), sodium acetate, and acetic acid were supplied by Sigma-Aldrich Corporation.

### 2.2 Preparation of Buffer Solutions

According to Table S1 (Supporting Information), 0.1 M acetate buffer solutions were prepared, and their pH values were confirmed using a 3051 Jenway pH meter.

### 2.3 Color Characterization

The color characteristics of resulting solutions were performed via CIE L*a*b* (CIELAB), which is a color space defined by the International Commission on Illumination (CIE) in 1976.^27,28^ It expresses color as three values: L* for the lightness from black (0) to white (100), a* from green (−) to red (+), and b* from blue (−) to yellow (+). In CIELAB color space, the color difference (ΔE*) is determined by calculating each of three values, which is expressed by eq 1(20)
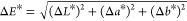
1where ΔE* ≈ 2.3 corresponds to a just visually noticeable difference.

Color measurement, reported in this paper, involved pipetting the sample aliquot (diluted 15 times using water) into a 1 cm poly(methyl methacrylate) (PMMA) plastic cuvette. Then, a DataColor CHECK 3 (DataColor Inc., U.K.), with an 8° diffuse D65 illuminant and at a 10° observer angle, was calibrated using a standard white and black plate and used to record samples’ L*, a*, and b* values against a white background. Each test was carried out in triplicate, and the mean value was reported.

### 2.4 High-Performance Liquid Chromatography (HPLC)

Analytical HPLC with diode array detection (DAD) was carried out using a reverse-phase C_18_ column and a water–acetonitrile gradient (acetonitrile: 5–50% within 5 min). The samples were diluted with water, and the concentration of samples was 1 mg/mL. The injection volume was 1 μL. The chosen wavelengths for detection were 254, 210, and 280 nm.

### 2.5 Experimental Design

#### 2.5.1 Selection of Factors and Their Ranges of Variation

The concentrations of DHA in sunless tanning products usually range from 1 to 10%.^3^ According to the previous study, 0.9 mol/L (≈9%) showed noticeable color formation. As such, the concentration of DHA was fixed at 0.9 mol/L in this study.

It has been reported that Arg, His, and Lys are abundant in SC and have relatively high reactivities with DHA. Boc-Arg-OH, Boc-His-OH, and Boc-Lys-OH are chosen to investigate the reactivity of side-chain amino groups and compare the color difference for reasons stated in Section 1 of this paper.

A total of six types of AA were experimentally studied: Arg, Boc-Arg-OH; His, Boc-His-OH; and Lys, Boc-Lys-OH. Based on the previous study, the parameters and their ranges of variation were chosen for the study reported here:

- Reaction time: 24, 48, and 72 h (three levels of variation)
- pH of the reaction mixture: 4.4, 5.0, and 5.6 (three levels of variation)
- Reaction temperature: 36, 43, and 50 °C (three levels of variation).

#### 2.5.2 Design of Factorial Experiments

Model Maillard reaction systems were designed through a set of full factorial experiments (DOE) using Minitab software. A set of 81 experiments (three factors, three levels, and three repetitions: 3^3^ × 3) for each amino acid were designed as shown in Tables S2–S7 (Supporting Information). To minimize the total number of experiments, the “amino acid type” was excluded from the full factorial design. Instead, the same 3^3^ × 3 design was applied to each of six AAs. As such, the total experiments conducted were 81 × 6 = 486.

### 2.6 Preparation of AA-DHA and Boc-AA-DHA Reaction Solutions

The six model reaction solutions were denoted as Arg-DHA (A-D), His-DHA (H-D), Lys-DHA (L-D), Boc-Arg-DHA (B-A-D), Boc-His-DHA (B-H-D), and Boc-Lys-DHA (B-L-D). These AA-DHA and Boc-AA-DHA solutions with the same molar ratio were prepared under different reaction conditions, as shown in Table S8 (Supporting Information). Thus, each sample was dissolved in 10 mL of 0.1 M acetate buffer solution (pH 4.4, 5.0, and 5.6) in a plastic test tube, sealed, and allowed to react at 36, 43, and 50 °C for 24, 48, and 72 h, respectively, according to the experimental design indicated in Tables S2–S7 (Supporting Information).

### 3 Results and Discussion

Although the absorbance at 420–450 nm has been extensively employed to evaluate the browning intensity of the Maillard reaction in food science, it is difficult to quantitatively describe color characteristics and changes, especially in dermato-cosmetic studies.^29^ Indeed, tristimulus colorimetry is a recommended and better approach to present the lightness, chroma, and hue by referring to the L*, a*, and b* values. The CIELAB color space, as a three-dimensional and uniform space, has been widely used in textiles, coatings, and cosmetics since its introduction in 1976.^30,31^ It not only covers the entire color range of human eye but also evaluates quantitatively the color difference (ΔE*) to control the color quality, as shown in eq.^32^ Therefore, the degree of browning and color characteristics of model systems reported in this study were mainly determined with the use of ΔE*, a*, and b* values.

### 3.1 Analysis of Color Difference of AA-DHA and Boc-AA-DHA Full Factorial Design of Experiments

Factorial design of experiment (DOE), as a widely used tool in the academia and industry, has been proven not only to analyze significant single factors but also to provide information about their interactions among factors, which are not possible to detect and identify with the traditional one-factor-at-a-time method.^33,34^ To better investigate the effects of reaction time, pH, and temperature on the color development, a full three-factors-three-levels with three repetitions (total 3^3^ × 3 = 81) experiment was designed for each amino acid–DHA tanning system. The analysis of variance (ANOVA) is a statistical method to estimate and test the main and interaction effects and to evaluate the reliability of the model.^35^ The *P*-value (*P* < 0.05) was adapted to determine whether the effect of the associated factor/interaction was significant or not. The smaller the *P*-value, the more significant the factor is.^36^ The *F*-value was employed to show how obviously a given factor affects the studied response, in conjunction with the *P*-value. The corresponding ANOVA results are summarized in Tables S9–S15 (Supporting Information).

**Figure 2:**
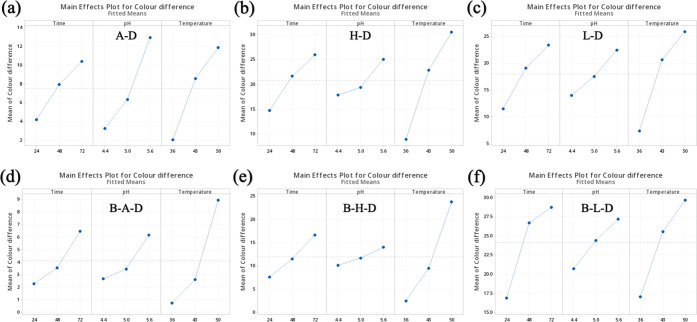
Main effect plots of reaction time, pH, and temperature on the color difference of (a) A-D, (b) H-D, (c) L-D, (d) B-A-D, (e) B-H-D, and (f) B-L-D.

The ANOVA results of all systems obtained showed that the main factors, reaction time, pH, and temperature, and some interaction effects were highly significant. The main effects represent deviations of the average between high and low levels for each one of them. Indeed, all main effects had a positive impact on the response. Thus, as the reaction time, pH, and temperature increased, the ΔE* values of all reaction systems showed an upward trend. The temperature was found to have the most significant influence on the ΔE*, followed by the reaction time, and finally pH in all reaction systems, except in A-D, according to the *F*-value shown in Tables S10–S15 (Supporting Information). Besides, the 2-way and 3-way interactions were significant, indicating that the influences of the factors studied on the ΔE* were dependent on each other. Meanwhile, temperature played a dominant role in the interactive effects of these factors. These phenomena can be seen intuitively in Figures 2 and S1 (Supporting Information), where the reaction time of 72 h, pH of 5.6, and temperature of 50 °C were considered to be the optimal reaction conditions to generate the deepest color. In addition, the mean ΔE* values of A-D (around 7.5) and H-D (around 21) were higher than those of B-A-D (around 4.1) and B-H-D (around 12). This is very understandable because the α-amino groups in A-D and H-D are conducive to the nucleophilic attack on the carbonyl groups of DHA, thus producing more colored compounds through the Schiff base intermediate. Besides, it has been reported that there are multiple routes to melanoidin production for 2° or higher amine.^37^ This can be used to explain that although the browning intensities of B-A-D and B-H-D are lower than those of A-D and B-D, they can still generate color through the reaction of a side-chain amine with DHA in different pathways. However, to our surprise, the mean ΔE* value of B-L-D (around 24) exhibited a higher value than that of L-D (around 18). To explore whether Boc-protected α-amino groups can form different melanoidins through other pathways leading to color deepening, Boc-Gly-OH and Boc-Ala-OH were selected to react with DHA under the same reaction conditions as L-D and B-L-D. This method that blocks the α-amine with Boc would completely remove that amine from the reaction pathway. It was found that the resulting solutions were colorless and did not generate color even at a higher temperature (60 °C) or in anhydrous solvents, such as dimethylformamide (DMF) and dimethyl sulfoxide (DMSO) (unpublished observations). These results suggested that the browning intensity of DHA tanning does not necessarily intensify as the number of amino groups increases and the amino group (ε-NH_2_) of the side chain of lysine plays a more important role in the color development, compared with its amino group (α-NH_2_).

### 3.2 Effects of pH and Reaction Time on the Browning Color Difference of AA-DHA and Boc-AA-DHA

Many studies have shown that pH plays a critical role in the food Maillard reaction because it not only affects the reactivity of the amino group and sugar but also leads to the formation of different reaction pathways and products.^38,39^ However, few people have systematically studied the effect of pH on the tanning reaction between DHA and AA/Boc-AA at different stages of reactions, i.e., as the reaction time increases. The pH of the human skin usually ranges from pH 4.0 to pH 7.0, and in most cases, it is about a pH of 5.5.^19^ Considering this fact, the pH values investigated were set at 4.4, 5.0, and 5.6. Figure 2 shows the effects of pH vs reaction time on the ΔE* of DHA tanning reactions. The corresponding data are summarized in Tables S16–S24 (Supporting Information). The corresponding sample images are shown in Figures S2–S4 (Supporting Information).

**Figure 3:**
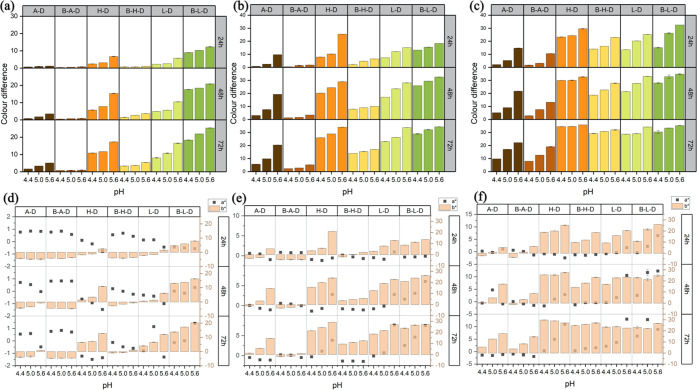
Effects of pH vs reaction time on the color difference and CIELAB of six model systems: (a, d) at 36 °C, (b, e) at 43 °C, and (c, f) at 50 °C.

As shown in Figure 3, the ΔE* values of the six systems showed an upward trend with the increase of pH and reaction time. The ΔE* values of H-D and L-D were always much higher than those of A-D. Such a phenomenon was due to the difference in the molecular structures of these AAs, thus producing different isoelectric points (pI) at 10.76, 7.59, and 9.74 for Arg, His, and Lys, respectively. At a given pH below their pI, AAs having low pI had relatively more unprotonated amino groups to facilitate nucleophilic attacks on the carbonyl groups of DHA and form more melanoidins. Besides, as the pH increased from 4.4 to 5.6, the value was still below their pI, but more unprotonated amino groups were released to react with DHA to produce more melanoidins, resulting in the increase of ΔE* value. Increases in both pH and reaction time significantly increase the color difference values of A-D and B-A-D, while they did not show an obvious effect on those of the remaining systems, especially at higher temperatures, such as 43 and 50 °C. For free AAs, the ΔE* values of H-D and L-D were always much higher than those of A-D, so did as Boc-AA.

In addition, to better understand the color characteristics of the AA/Boc-AA-DHA tanning reaction systems, the effects of pH and reaction time on a* (redness) and b* (yellowness) values were also investigated. As exhibited in Figure 3d–f, with the increase of pH and reaction time, the b* value shows a similar upward trend for the same system, suggesting that the color is getting increasingly yellow. The highest b* values of A-D, B-A-D, H-D, B-H-D, L-D, and B-L-D were achieved at 72h and pH 5.6 with the values of 0.81, −4.58, 12.80, 1.03, 12.10, and 20.31 for temperature 36 °C, 14.62, 0.91, 29.05, 12.37, 26.95, and 27.07 for temperature 43 °C, and 17.60, 14.73, 27.15, 27.13, 22.49, and 26.70 for temperature 50 °C, respectively. However, these changes have little effect on the value of a*. a* fluctuates significantly in different AA-DHA and Boc-AA-DHA systems and does not show a uniform change trend. These results indicated that the b* value played a major role in the ΔE* of the tanning reaction when its pH and reaction time were increased.

### 3.3 Effects of Temperature vs Reaction Time on the Browning Color Difference of AA-DHA and Boc-AA-DHA

**Figure 4:**
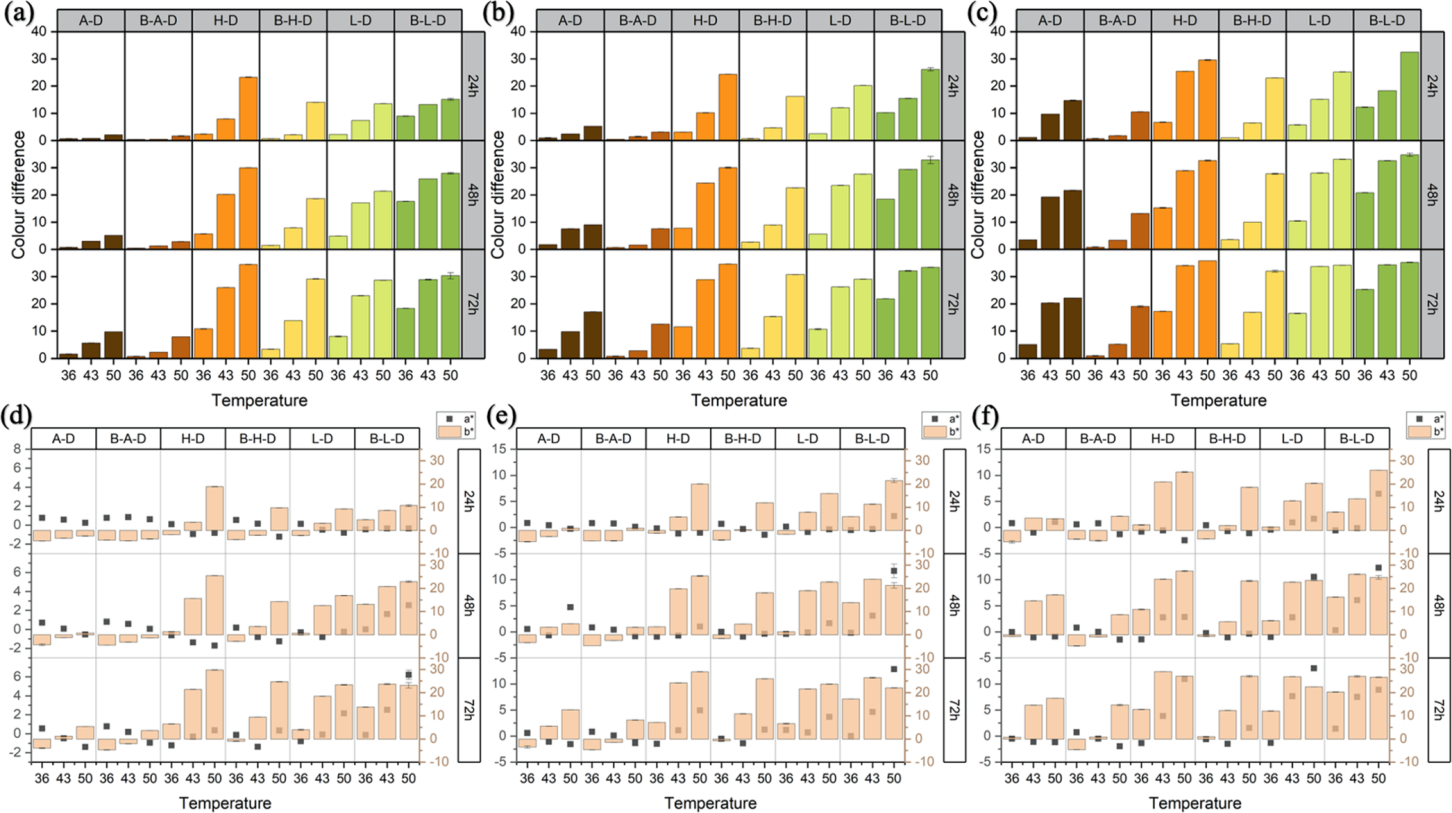
Effects of temperature vs reaction time on the color difference and CIELAB of six model systems: (a, d) at pH 4.4, (b, e) at pH 5.0, and (c, f) at pH 5.6.

As another important factor affecting the Maillard reaction, temperature has also been widely studied in the heat treatment of food at high temperatures (usually above 90 °C).^40,41^ However, the temperature of the DHA tanning reaction on the skin is much lower than that for food heating. The normal skin temperature is around 36 °C, and it can tolerate higher temperatures of no more than 50 °C without being harmed. Considering this fact, the temperature for this study was set at 36, 43, and 50 °C. The effects of temperature vs reaction time on the color difference of AA-DHA and Boc-AA-DHA systems are shown in Figure 4. The corresponding data are summarized in Tables S16–S24 (Supporting Information).

In general, a significant increase in ΔE* value for all systems was observed when the temperature increased from 36 to 50 °C at the same pH and reaction time. Researchers have confirmed the observation that an increase in temperature can promote the reaction rate and browning intensity of the Maillard reaction. The extent of increase in the ΔE* from 36 to 43 °C was much greater than that from 43 to 50 °C, which was consistent with the phenomenon observed from the temperature factorial plots in Figure 1. Besides, as the time increased from 24 to 72 h at a fixed pH, the effect of temperature on the ΔE* was not obvious, especially at 43 and 50 °C, for most systems. In terms of the browning intensity, for free AAs, the ΔE* values of H-D and L-D were still much higher than those of A-D and so were those of Boc-AA. The highest ΔE* values of A-D, B-A-D, H-D, B-H-D, L-D, and B-L-D were reached at 72h and 50 °C with the values of 9.78, 7.92, 34.48, 29.11, 28.67, and 30.32 for pH 4.4, 17.05, 12.60, 34.62, 30.70, 29.02, and 33.39 for pH 5.0, and 22.15, 19.07, 35.80, 31.96, 34.17, and 35.22 for pH 5.6, respectively. Figures 4d,e,f shows the variations in a* and b* with the increase of temperature vs reaction time. The b* values of all systems showed a similar upward trend as that of the color difference, while the trends of variation of the a* value still fluctuated. Unlike the trend of a* changes against the pH and reaction time in A-D, B-A-D, and B-H-D systems, for H-D, L-D, and B-L-D systems, the a* value started to show a uniform upward trend with the increase in temperature and reaction time, suggesting that the color difference at higher temperatures was attributed to the changes of a* and b* together.

Additionally, to further explore the reason why the color became darker with the increasing temperature, A-D, H-D, L-D, B-A-D, B-H-D, and B-L-D systems were examined using the analytical HPLC to compare the number and intensity of peaks appearing in the same system at different temperatures, as shown in Figures S5–S10 (Supporting Information). The HPLC chromatograms markedly showed that for the same AA-DHA and Boc-AA-DHA systems, the peak number and its eluting time were similar and the intensity of the same peak increased, indicating that higher temperatures did not result in the formation of different colored products but led to an increase in the concentration of the same or similar colored products, accompanied with the deepening color of the solution and the increase in the ΔE* value.

### 3.4 Comparison of Browning Color Differences between AA-DHA and Boc-AA-DHA Systems

**Figure 5:**
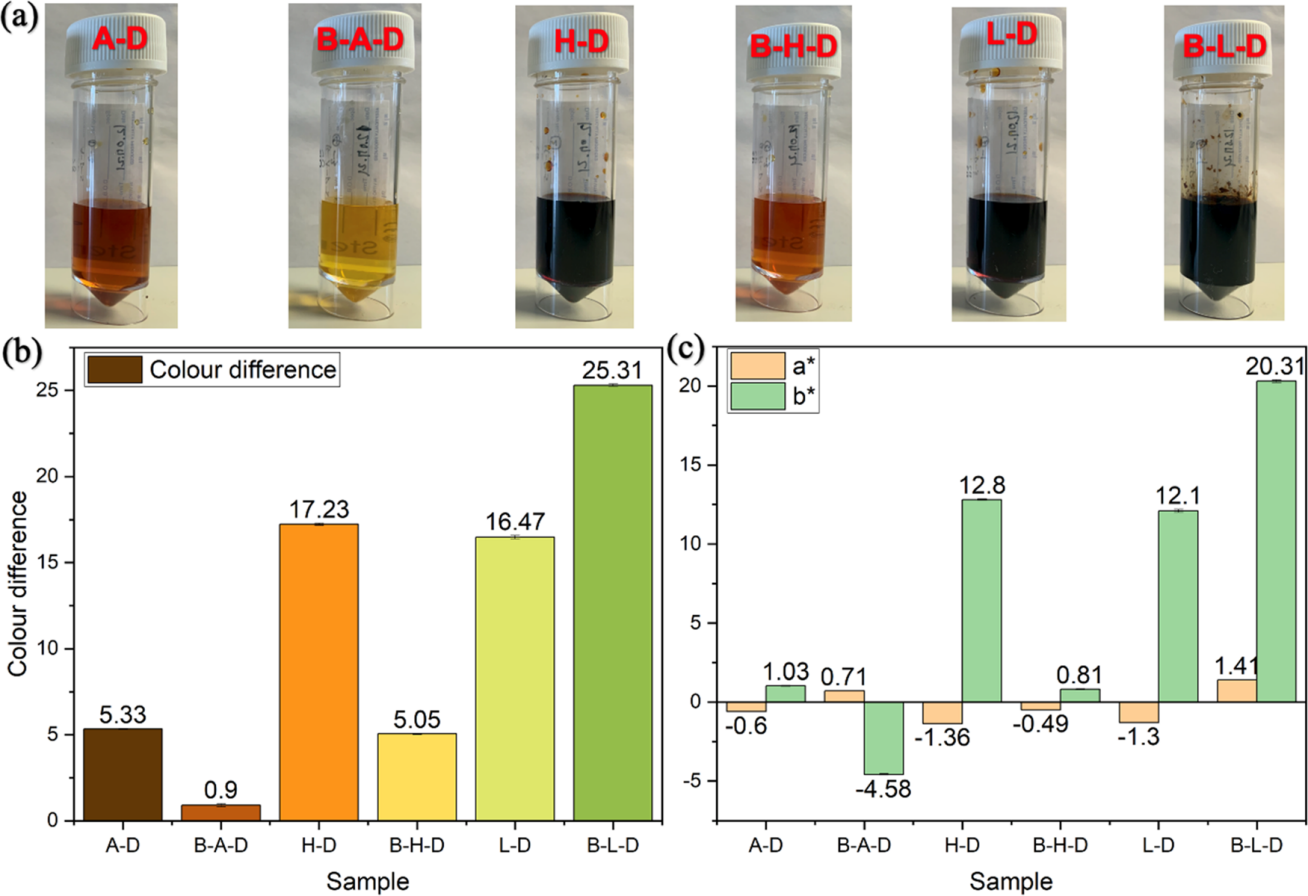
(a) Sample images, (b) color difference, and (c) a* and b* values of six model systems at 36 °C, pH 5.6, and 72 h.

It was observed that the color development is significantly affected by the types of AAs. Arg, Lys, and His are representative due to their high contents in the SC of human skin. The ΔE* values of A-D are much lower than those of H-D and L-D under the same reaction conditions. The phenomenon is linked to the pI and steric characteristics of AAs, and the corresponding explanation has been discussed in our previously published paper. In this section, we focus on discussing the differences between AAs and Boc-AAs in the color development. As shown in Figure 5, at 36 °C, pH 5.6, and 72 h, B-A-D was found to give the lowest ΔE* values of 0.9, compared with those of B-H-D (5.05) and B-L-D (25.31), which was the same as the A-D. It was postulated that Boc-AAs do not develop a stronger color than AAs because Boc-AAs lack an α-NH_2_ and possess only one primary or secondary amino group on the side chain that can react with DHA to form melanoidins. As expected, A-D and H-D have superior ΔE* values than those of B-A-D and B-H-D. However, interestingly, B-L-D shows larger ΔE* values than does L-D under any of the same conditions, although the difference becomes smaller at higher temperatures, e.g., 43 and 50 °C. Besides, the ΔE* value of B-L-D is also higher than that of B-H-D under the same reaction conditions, even greater than that of H-D in most cases, because the ε-amino group of lysine is less sterically hindered and more reactive than the imidazole of histidine. Even A-D has more amino groups in the molecule, but the color formation is not successful as B-H-D and B-L-D do. These results indicated that the color development depends on the structure and reactivity of the amino group, rather than the number of amino groups in these systems. It may be postulated that the high reactivity amino group affords easier and faster reactions to form more melanoidin with various chromophores.

To further validate this assumption, analytical HPLC was performed to detect and quantify the formed compounds, as shown in Figure S11. The HPLC chromatogram showed that the number of peaks for B-L-D was much greater than that for L-D under the same reaction conditions, e.g., B-L-D had 44 peaks and L-D had 23 peaks at 254 nm when reacted for 72 h at pH 5.6 and 50 °C. Besides, it is worth noting that there was only one main peak (eluting at 0.74 min) that accounted for 31% of L-D at 254 nm, while three main peaks (eluting at 0.73, 1.32, and 3.49 min) were found for B-L-D at 254 nm, with the percentage compositions being 15, 9, and 7%, respectively. This implies that these peaks may represent key colored compounds that explain the phenomenon that the color of B-L-D was darker than that of L-D. However, further studies will be required to identify the exact chemical structures, chromophores, and color intensity of these key colored compounds.

## 4 Conclusions

In summary, the color development kinetics, including the extent of browning and color characteristics, of all tanning reactions were effectively evaluated by CIELAB, such as the ΔE*, a*, and b* values. The factorial experiment results showed that the factors (reaction time, pH, and temperature) and their interactions were significant and had a positive impact on the browning intensity. As the reaction time, pH, and temperature increased, the ΔE* values of all systems showed an upward trend, mainly reflected in the change of yellowness (b*). The temperature was found to have the most significant influence on the ΔE* and play a dominant role in the interactions with the reaction time and pH. The b* values demonstrated a similar upward trend as that of ΔE*, while the variation of a* values did not follow a constant trend. Only H-D, L-D, and B-L-D were observed to exhibit a uniform upward trend with the increased temperature and reaction time. The higher the temperature, the more pronounced the phenomenon. In addition, the color development was significantly affected by the types of AAs. Arg and His reacted with DHA more rapidly than Boc-Arg and Boc-His, as expected. However, interestingly, Boc-Lys developed a stronger color than Lys at any set of the same reaction conditions, suggesting that ε-NH_2_ of the side chain of lysine, appearing in peptides or proteins of skin, may play a more important role in the color development of DHA tanning. Even though A-D has more amino groups in the molecule, its color formation was not as successful as B-H-D and B-L-D. These results indicated that the color development depends on the structure and reactivity of the amino group, rather than its number in these systems. In the subsequent studies, the authors will isolate and identify key colored compounds formed in these systems and compare their color intensities with different chromophores, aiming to further understand the color development mechanism of DHA tanning on human skin.

## Supporting Information Available

Design and analysis of factorial experiments (Tables S1–S15 and Figure S1); CIELAB data (Tables S16–S24); sample images (Figures S2–S4); and analytical HPLC results (Figures S5–S11) (PDF)

## References

[ref1] Burkhart C. G.; Burkhart C. N. Dihydroxyacetone and methods to improve its performance as artificial tanner. Open Dermatol. J. 2009, 3, 42–43. 10.2174/1874372200903010042.

[ref2] Ciriminna R.; Fidalgo A.; Ilharco L. M.; Pagliaro M. Dihydroxyacetone: An updated insight into an important bioproduct. ChemistryOpen 2018, 7, 233–236. 10.1002/open.201700201. 29531886 PMC5838383

[ref3] Garone M.; Howard J.; Fabrikant J. A review of common tanning methods. J. Clin. Aesthet. Dermatol. 2015, 8, 43–47. PMC4345932 25741402

[ref4] Fisher D. E.; James W. D. Indoor tanning - science, behavior, and policy. N. Engl. J. Med. 2010, 363, 901–903. 10.1056/NEJMp1005999. 20818900 PMC3951814

[ref5] Stapleton J. L.; Hillhouse J. Industry influence in indoor tanning research. BMJ 2020, 368, m345 10.1136/bmj.m345. 32019763

[ref6] Faurschou A.; Wulf H. C. Durability of the sun protection factor provided by dihydroxyacetone. Photodermatol., Photoimmunol. Photomed. 2004, 20, 239–242. 10.1111/j.1600-0781.2004.00118.x. 15379873

[ref7] Munt D. J.; Grana A.; Hulce M.; Fusaro R. M.; Dash A. K. Effect of simultaneous administration of dihydroxyacetone on the diffusion of lawsone through various in Vitro skin models. AAPS PharmSciTech 2015, 16, 1425–1433. 10.1208/s12249-015-0335-8. 25986597 PMC4666250

[ref8] Garcia A. C.; Kolb M. J.; van Nierop y Sanchez C.; Vos J.; Birdja Y. Y.; Kwon Y.; Tremiliosi-Filho G.; Koper M. T. M. Strong impact of platinum surface structure on primary and secondary alcohol oxidation during electro-oxidation of glycerol. ACS Catal. 2016, 6, 4491–4500. 10.1021/acscatal.6b00709.

[ref9] Zheng Z.; Luo M.; Yu J.; Wang J.; Ji J. Novel process for 1,3-dihydroxyacetone production from glycerol. 1. Technological feasibility study and process design. Ind. Eng. Chem. Res. 2012, 51, 3715–3721. 10.1021/ie201710h.

[ref10] Martins S. I. F. S.; Jongen W. M. F.; Van Boekel M. A. J. S. A review of Maillard reaction in food and implications to kinetic modelling. Trends Food Sci. Technol. 2000, 11, 364–373. 10.1016/S0924-2244(01)00022-X.

[ref11] Somoza V.; Fogliano V. 100 years of the maillard reaction: Why our food turns brown. J. Agric. Food Chem. 2013, 61, 10197 10.1021/jf403107k. 24107120

[ref12] Wittgenstein E.; Berry H. K. Staining of skin with dihydroxyacetone. Science 1960, 132, 894–895. 10.1126/science.132.3431.894. 13845496

[ref13] Ricapito N. G.; Ghobril C.; Zhang H.; Grinstaff M. W.; Putnam D. Synthetic biomaterials from metabolically derived synthons. Chem. Rev. 2016, 116, 2664–2704. 10.1021/acs.chemrev.5b00465. 26821863 PMC5810137

[ref14] Nguyen B. C.; Kochevar I. E. Factors influencing sunless tanning with dihydroxyacetone. Br. J. Dermatol. 2003, 149, 332–340. 10.1046/j.1365-2133.2003.05434.x. 12932240

[ref15] Huang A.; Brody N.; Liebman T. N. Dihydroxyacetone and sunless tanning: Knowledge, myths, and current understanding. J. Am. Acad. Dermatol. 2017, 77, 991–992. 10.1016/j.jaad.2017.04.1117. 29029917

[ref16] Matsunaga K.; Sasaki M.; Okajima T.; Miyaki M.; Sakaguchi H. Improvement in the quality of life of patients with rhododendrol-induced leukoderma after camouflaging with dihydroxyacetone cream. J. Dermatol. 2020, 47, 801–802. 10.1111/1346-8138.15398. 32424834 PMC7383915

[ref17] Rajatanavin N.; Suwanachote S.; Kulkollakarn S. Dihydroxyacetone: A safe camouflaging option in vitiligo. Int. J. Dermatol. 2008, 47, 402–406. 10.1111/j.1365-4632.2008.03356.x. 18377610

[ref18] Lustig B.; Katchen B.; Reiss F. The amino acid composition of the horny layer of the human skin. J. Invest. Dermatol. 1958, 30, 159–163. 10.1038/jid.1958.29. 13525788

[ref19] Sun Y.; Lin L.; Zhang P. Color development kinetics of Maillard reactions. Ind. Eng. Chem. Res. 2021, 60, 3495–3501. 10.1021/acs.iecr.1c00026.

[ref20] Nguyen B. C.; Kochevar I. E. Influence of hydration on dihydroxyacetone-induced pigmentation of stratum corneum. J. Invest. Dermatol. 2003, 120, 655–661. 10.1046/j.1523-1747.2003.12089.x. 12648231

[ref21] Hellwig M.; Henle T. Baking, ageing, diabetes: A short history of the Maillard reaction. Angew. Chem., Int. Ed. 2014, 53, 10316–10329. 10.1002/anie.201308808. 25044982

[ref22] Kamalov M.; Harris P. W. R.; Wood J. M.; Brimble M. A. On resin synthesis and cross-linking of collagen peptides containing the advanced glycation end-product pyrraline via Maillard condensation. Chem. Commun. 2015, 51, 9475–9478. 10.1039/c5cc03052h. 25963401

[ref23] Kaur H.; Kamalov M.; Brimble M. A. Chemical synthesis of peptides containing site-specific advanced glycation endproducts. Acc. Chem. Res. 2016, 49, 2199–2208. 10.1021/acs.accounts.6b00366. 27672697

[ref24] Sjoblom N. M.; Kelsey M. M. G.; Scheck R. A. A systematic study of selective protein glycation. Angew. Chem. 2018, 130, 16309–16314. 10.1002/ange.201810037. 30290036

[ref25] Lin J. A.; Wu C. H.; Yen G. C. Perspective of advanced glycation end products on human health. J. Agric. Food Chem. 2018, 66, 2065–2070. 10.1021/acs.jafc.7b05943. 29421872

[ref26] McEwen J. M.; Fraser S.; Guir A. L. S.; Dave J.; Scheck R. A. Synergistic sequence contributions bias glycation outcomes. Nat. Commun. 2021, 12, 3316 10.1038/s41467-021-23625-8. 34083524 PMC8175500

[ref27] Robertson A. R. The CIE 1976 color-difference formulae. Color Res. Appl. 1977, 2, 7–11. 10.1002/j.1520-6378.1977.tb00104.x.

[ref28] Brill M. H. Is CIELAB one space or many?. Color. Technol. 2021, 137, 83–85. 10.1111/cote.12486.

[ref29] Hong P. K.; Betti M. Non-enzymatic browning reaction of glucosamine at mild conditions: Relationship between colour formation, radical scavenging activity and α-dicarbonyl compounds production. Food Chem. 2016, 212, 234–243. 10.1016/j.foodchem.2016.05.170. 27374528

[ref30] Fan Y.; Zhang Y. Q.; Yan K.; Long J. J. Synthesis of a Novel Disperse Reactive Dye Involving a Versatile Bridge Group for the Sustainable Coloration of Natural Fibers in Supercritical Carbon Dioxide. Adv. Sci. 2019, 6, 1801368 10.1002/advs.201801368. PMC6325576 30643724

[ref31] Amano K.; Xiao K.; Wuerger S.; Meyer G. A colorimetric comparison of sunless with natural skin tan. PLoS One 2020, 15, e0233816 10.1371/journal.pone.0233816. 33315862 PMC7735640

[ref32] Egurrola G.; García J.; Segura J. A new analytical method for the evaluation of cosmetic pigments. Color Res. Appl. 2021, 46, 821–829. 10.1002/col.22621.

[ref33] Azeez S.; Lasekan O.; Jinap S.; Sulaiman R. Effect of roasting conditions on the browning intensity and structural changes in jackfruit (Artocarpus hetrophyllus) seeds. J. Food Sci. Technol. 2015, 52, 8050–8058. 10.1007/s13197-015-1900-6. 26604377 PMC4648944

[ref34] Savic S. M.; Cekic N. D.; Savic S. R.; Ilic T. M.; Savic S. D. ‘All-natural’ anti-wrinkle emulsion serum with Acmella oleracea extract: A design of experiments (DoE) formulation approach, rheology and in vivo skin performance/efficacy evaluation. Int. J. Cosmet. Sci. 2021, 43, 530–546. 10.1111/ics.12726. 34297422

[ref35] Nakhaie D.; Kosari A.; Mol J. M. C.; Asselin E. Corrosion resistance of hot-dip galvanized steel in simulated soil solution: A factorial design and pit chemistry study. Corros. Sci. 2020, 164, 108310 10.1016/j.corsci.2019.108310.

[ref36] Bruno Siewe F.; Kudre T. G.; Narayan B. Optimisation of ultrasound-assisted enzymatic extraction conditions of umami compounds from fish by-products using the combination of fractional factorial design and central composite design. Food Chem. 2021, 334, 127498 10.1016/j.foodchem.2020.127498. 32688179

[ref37] Carnali J. O.; Madison S. A.; Shah P.; Qiu Q. Structure/property relationship for ethylenediamine derivatives as aids in sunless tanning. Ind. Eng. Chem. Res. 2012, 51, 15573–15581. 10.1021/ie301929w.

[ref38] Pham H. T. T.; Kityo P.; Buvé C.; Hendrickx M. E.; Van Loey A. M. Influence of pH and composition on nonenzymatic browning of shelf-stable orange juice during storage. J. Agric. Food Chem. 2020, 68, 5402–5411. 10.1021/acs.jafc.9b07630. 32302128

[ref39] Geng J. T.; Takahashi K.; Kaido T.; Kasukawa M.; Okazaki E.; Osako K. Relationship among pH, generation of free amino acids, and Maillard browning of dried Japanese common squid Todarodes pacificus meat. Food Chem. 2019, 283, 324–330. 10.1016/j.foodchem.2019.01.056. 30722879

[ref40] Stadler R. H.; Blank I.; Varga N.; Robert F.; Hau J.; Guy P. A.; Robert M.-C.; Riediker S. Acrylamide from Maillard reaction products. Nature 2002, 419, 449–450. 10.1038/419449a. 12368845

[ref41] Cui H.; Yu J.; Zhai Y.; Feng L.; Chen P.; Hayat K.; Xu Y.; Zhang X.; Ho C. T. Formation and fate of Amadori rearrangement products in Maillard reaction. Trends Food Sci. Technol. 2021, 115, 391–408. 10.1016/j.tifs.2021.06.055.

