# Supplementary material for: Comparison of Color Development Kinetics of Tanning Reactions of Dihydroxyacetone with Free and Protected Basic Amino Acids
Source: ACS Omega. 2022 Dec 1;7(49):45510–7. doi: 10.1021/acsomega.2c06124 (PMC9753197; doi:10.1021/acsomega.2c06124)
Supplement: Supplementary file 1 — ao2c06124_si_001.pdf [file ao2c06124_si_001.pdf]

Supporting Information

Comparison of colour development kinetics of tanning reactions of dihydroxyacetone with free and protected basic amino acids

Yufa Sun, Subin Lee and Long Lin*

Colour Science, School of Chemistry, University of Leeds, Woodhouse Lane, Leeds LS2 9JT, UK

*Corresponding email: l.lin@leeds.ac.uk; linlongprofessor@outlook.com

Table of Contents

Table S1. Preparation of acetate buffer with different pH values **S4**

Table S2. Factorial design to study the effects of reaction time, pH and temperature on the tanning reaction of A-D **S4**

Table S3. Factorial design to study the effects of reaction time, pH and temperature on the tanning reaction of B-A-D **S7**

Table S4. Factorial design to study the effects of reaction time, pH and temperature on the tanning reaction of H-D **S9**

Table S5. Factorial design to study the effects of reaction time, pH and temperature on the tanning reaction of B-H-D **S11**

Table S6. Factorial design to study the effects of reaction time, pH and temperature on the tanning reaction of L-D **S13**

Table S7. Factorial design to study the effects of reaction time, pH and temperature on the tanning reaction of B-L-D **S15**

Table S8. Preparation of AA-DHA for studying the effects of reaction time, pH and temperature on the tanning reaction **S17**

Table S9. Model summary of factorial design of experiment (DOE) **S17**

Table S10. The analysis of variance (ANOVA) of A-D **S17**

Table S11. The analysis of variance (ANOVA) of B-A-D **S18**

Table S12. The analysis of variance (ANOVA) of H-D **S18**

Table S13. The analysis of variance (ANOVA) of B-H-D **S19**

Table S14. The analysis of variance (ANOVA) of L-D **S19**

Table S15. The analysis of variance (ANOVA) of B-L-D **S20**

Figure S1. Interaction plots of reaction time, pH and temperature on the colour difference of (a) A-D (b) H-D (c) L-D (d) B-A-D (e) B-H-D and (f) B-L-D **S20**

Table S16. CIELAB results of six model systems obtained at pH 4.4 and 36 °C **S21**

Table S17. CIELAB results of six model systems obtained at pH 5.0 and 36 °C **S21**

Table S18. CIELAB results of six model systems obtained at pH 5.6 and 36 °C **S22**

Table S19. CIELAB results of six model systems obtained at pH 4.4 and 43 °C **S22**

Table S20. CIELAB results of six model systems obtained at pH 5.0 and 43 °C **S23**

Table S21. CIELAB results of six model systems obtained at pH 5.6 and 43 °C **S23**

Table S22. CIELAB results of six model systems obtained at pH 4.4 and 50°C **S24**

Table S23. CIELAB results of six model systems obtained at pH 5.0 and 50 °C **S24**

Table S24. CIELAB results of six model systems obtained at pH 5.6 and 50°C **S25**

Figure S2. Sample images of A-D and B-A-D at 72h with varying pH and temperature: (a) A-D, 36 °C (b) A-D, 43°C (c) A-D, 50°C (d) B-A-D, 36°C (e) B-A-D, 43°C and (f) B-A-D, 50 °C ***S25***

Figure S3. Sample images of H-D and B-H-D at 72h with varying pH and temperature: (a) H-D, 36 °C (b) H-D, 43°C (c) H-D, 50°C (d) B-H-D, 36°C (e) B-H-D, 43°C and (f) B-H-D, 50 °C ***S26***

Figure S4. Sample images of L-D and B-L-D at 72h with varying pH and temperature: (a) L-D, 36 °C (b) L-D, 43°C (c) L-D, 50°C (d) B-L-D, 36°C (e) B-L-D, 43°C and (f) B-L-D, 50 °C . ***S26***

Figure S5. Analytical HPLC of A-D at (a) 72 h, pH 5.6, 43 °C and (b) 72 h, pH 5.6, 50 °C (1 µL, rapid 5-50% MeCN, 5 min, signals at 254, 210 and 280 nm).. ***S27***

Figure S6. Analytical HPLC of H-D at (a) 72 h, pH 5.6, 43 °C and (b) 72 h, pH 5.6, 50 °C (1 µL, rapid 5-50% MeCN, 5 min, signals at 254, 210 and 280 nm).. ***S27***

Figure S7. Analytical HPLC of L-D at (a) 72 h, pH 5.6, 43 °C and (b) 72 h, pH 5.6, 50 °C (1 µL, rapid 5-50% MeCN, 5 min, signals at 254, 210 and 280 nm).. ***S28***

Figure S8. Analytical HPLC of B-A-D at (a) 72 h, pH 5.6, 43 °C and (b) 72 h, pH 5.6, 50 °C (1 µL, rapid 5-50% MeCN, 5 min, signals at 254, 210 and 280 nm) ***S28***

Figure S9. Analytical HPLC of B-H-D at (a) 72 h, pH 5.6, 43 °C and (b) 72 h, pH 5.6, 50 °C (1 µL, rapid 5-50% MeCN, 5 min, signals at 254, 210 and 280 nm) ***S29***

Figure S10. Analytical HPLC of B-L-D at (a) 72 h, pH 5.6, 43 °C and (b) 72 h, pH 5.6, 50 °C (1 µL, rapid 5-50% MeCN, 5 min, signals at 254, 210 and 280 nm) ***S29***

Figure S11. Analytical HPLC of (a) L-D at 72 h, pH 5.6, 50 °C and (b) B-L-D at 72 h, pH 5.6, 50 °C (1 µL, rapid 5-50% MeCN, 5 min, signals at 254, 210 and 280 nm) ***S30***

Table S1. Preparation of acetate buffer with different pH values

pH	Sodium acetate (g)	Acetic acid (g)	H_2O (mL)
**4.4**	1.663	1.785	500
**5.0**	2.762	0.981	500
**5.6**	3.861	0.176	500

Table S2. Factorial design to study the effects of reaction time, pH and temperature on the tanning reaction of A-D

Std Order	Run Order	Pt Type	Block	Time	pH	Temp	Colour Difference
1	46	1	1	24	4.4	36	0.62
2	4	1	1	24	4.4	43	0.81
3	33	1	1	24	4.4	50	2.03
4	75	1	1	24	5	36	0.84
5	49	1	1	24	5	43	2.41
6	80	1	1	24	5	50	5.25
7	17	1	1	24	5.6	36	1.15
8	5	1	1	24	5.6	43	9.73
9	2	1	1	24	5.6	50	14.82
10	37	1	1	48	4.4	36	0.78
11	14	1	1	48	4.4	43	3.00
12	58	1	1	48	4.4	50	5.15
13	64	1	1	48	5	36	1.81
14	11	1	1	48	5	43	7.58
15	56	1	1	48	5	50	9.03
16	71	1	1	48	5.6	36	3.48
17	65	1	1	48	5.6	43	19.25
18	21	1	1	48	5.6	50	21.66
19	69	1	1	72	4.4	36	1.49
20	20	1	1	72	4.4	43	5.60
21	53	1	1	72	4.4	50	9.78
22	39	1	1	72	5	36	3.29
23	23	1	1	72	5	43	9.84
24	44	1	1	72	5	50	17.05
25	19	1	1	72	5.6	36	5.01
26	73	1	1	72	5.6	43	19.14
27	60	1	1	72	5.6	50	22.16
28	43	1	1	24	4.4	36	0.75

29	7	1	1	24	4.4	43	0.80
30	1	1	1	24	4.4	50	2.06
31	31	1	1	24	5	36	1.04
32	41	1	1	24	5	43	2.42
33	48	1	1	24	5	50	5.22
34	77	1	1	24	5.6	36	1.19
35	54	1	1	24	5.6	43	9.73
36	6	1	1	24	5.6	50	14.82
37	55	1	1	48	4.4	36	0.81
38	61	1	1	48	4.4	43	3.01
39	50	1	1	48	4.4	50	5.14
40	78	1	1	48	5	36	1.80
41	42	1	1	48	5	43	7.59
42	45	1	1	48	5	50	9.04
43	67	1	1	48	5.6	36	3.43
44	16	1	1	48	5.6	43	19.21
45	18	1	1	48	5.6	50	21.70
46	51	1	1	72	4.4	36	1.57
47	28	1	1	72	4.4	43	5.65
48	35	1	1	72	4.4	50	9.79
49	30	1	1	72	5	36	3.30
50	24	1	1	72	5	43	9.85
51	57	1	1	72	5	50	17.10
52	36	1	1	72	5.6	36	5.09
53	76	1	1	72	5.6	43	19.18
54	62	1	1	72	5.6	50	22.14
55	47	1	1	24	4.4	36	0.71
56	38	1	1	24	4.4	43	0.78
57	10	1	1	24	4.4	50	2.15
58	3	1	1	24	5	36	0.96
59	59	1	1	24	5	43	2.43
60	70	1	1	24	5	50	5.22
61	79	1	1	24	5.6	36	1.08
62	74	1	1	24	5.6	43	9.74
63	29	1	1	24	5.6	50	14.59
64	63	1	1	48	4.4	36	0.72
65	27	1	1	48	4.4	43	3.00
66	72	1	1	48	4.4	50	5.14
67	40	1	1	48	5	36	1.82
68	81	1	1	48	5	43	7.59
69	15	1	1	48	5	50	9.02
70	12	1	1	48	5.6	36	3.46
71	66	1	1	48	5.6	43	19.16
72	52	1	1	48	5.6	50	21.68

73	8	1	1	72	4.4	36	1.56
74	25	1	1	72	4.4	43	5.69
75	13	1	1	72	4.4	50	9.77
76	68	1	1	72	5	36	3.27
77	9	1	1	72	5	43	9.82
78	26	1	1	72	5	50	17.02
79	34	1	1	72	5.6	36	5.05
80	32	1	1	72	5.6	43	19.21
81	22	1	1	72	5.6	50	22.16

Table S3. Factorial design to study the effects of reaction time, pH and temperature on the tanning reaction of B-A-D

Std Order	Run Order	Pt Type	Block	Time	pH	Temp	Colour Difference
1	46	1	1	24	4.4	36	0.38
2	4	1	1	24	4.4	43	0.46
3	33	1	1	24	4.4	50	1.60
4	75	1	1	24	5	36	0.49
5	49	1	1	24	5	43	1.43
6	80	1	1	24	5	50	3.16
7	17	1	1	24	5.6	36	0.72
8	5	1	1	24	5.6	43	2.00
9	2	1	1	24	5.6	50	10.54
10	37	1	1	48	4.4	36	0.50
11	14	1	1	48	4.4	43	1.34
12	58	1	1	48	4.4	50	2.84
13	64	1	1	48	5	36	0.62
14	11	1	1	48	5	43	1.69
15	56	1	1	48	5	50	7.58
16	71	1	1	48	5.6	36	0.84
17	65	1	1	48	5.6	43	3.38
18	21	1	1	48	5.6	50	13.19
19	69	1	1	72	4.4	36	1.49
20	20	1	1	72	4.4	43	5.60
21	53	1	1	72	4.4	50	9.78
22	39	1	1	72	5	36	0.80
23	23	1	1	72	5	43	2.75
24	44	1	1	72	5	50	12.58
25	19	1	1	72	5.6	36	0.90
26	73	1	1	72	5.6	43	5.18
27	60	1	1	72	5.6	50	18.81
28	43	1	1	24	4.4	36	0.37
29	7	1	1	24	4.4	43	0.46
30	1	1	1	24	4.4	50	1.65
31	31	1	1	24	5	36	0.48
32	41	1	1	24	5	43	1.48
33	48	1	1	24	5	50	3.17
34	77	1	1	24	5.6	36	0.70
35	54	1	1	24	5.6	43	1.79
36	6	1	1	24	5.6	50	10.55
37	55	1	1	48	4.4	36	0.49
38	61	1	1	48	4.4	43	1.30
39	50	1	1	48	4.4	50	2.83
40	78	1	1	48	5	36	0.79

41	42	1	1	48	5	43	1.67
42	45	1	1	48	5	50	7.61
43	67	1	1	48	5.6	36	0.86
44	16	1	1	48	5.6	43	3.38
45	18	1	1	48	5.6	50	13.18
46	51	1	1	72	4.4	36	1.57
47	28	1	1	72	4.4	43	5.65
48	35	1	1	72	4.4	50	9.79
49	30	1	1	72	5	36	0.72
50	24	1	1	72	5	43	2.77
51	57	1	1	72	5	50	12.59
52	36	1	1	72	5.6	36	0.81
53	76	1	1	72	5.6	43	5.14
54	62	1	1	72	5.6	50	19.03
55	47	1	1	24	4.4	36	0.40
56	38	1	1	24	4.4	43	0.50
57	10	1	1	24	4.4	50	1.77
58	3	1	1	24	5	36	0.49
59	59	1	1	24	5	43	1.44
60	70	1	1	24	5	50	3.15
61	79	1	1	24	5.6	36	0.70
62	74	1	1	24	5.6	43	1.61
63	29	1	1	24	5.6	50	10.55
64	63	1	1	48	4.4	36	0.47
65	27	1	1	48	4.4	43	1.27
66	72	1	1	48	4.4	50	2.85
67	40	1	1	48	5	36	0.79
68	81	1	1	48	5	43	1.69
69	15	1	1	48	5	50	7.58
70	12	1	1	48	5.6	36	0.89
71	66	1	1	48	5.6	43	3.36
72	52	1	1	48	5.6	50	13.16
73	8	1	1	72	4.4	36	1.56
74	25	1	1	72	4.4	43	5.69
75	13	1	1	72	4.4	50	9.77
76	68	1	1	72	5	36	0.81
77	9	1	1	72	5	43	2.78
78	26	1	1	72	5	50	12.61
79	34	1	1	72	5.6	36	0.99
80	32	1	1	72	5.6	43	5.17
81	22	1	1	72	5.6	50	19.38

Table S4. Factorial design to study the effects of reaction time, pH and temperature on the tanning reaction of H-D

Std Order	Run Order	Pt Type	Block	Time	pH	Temp	Colour Difference
1	46	1	1	24	4.4	36	2.42
2	4	1	1	24	4.4	43	7.89
3	33	1	1	24	4.4	50	23.41
4	75	1	1	24	5	36	3.06
5	49	1	1	24	5	43	10.08
6	80	1	1	24	5	50	24.34
7	17	1	1	24	5.6	36	6.54
8	5	1	1	24	5.6	43	25.46
9	2	1	1	24	5.6	50	29.48
10	37	1	1	48	4.4	36	5.67
11	14	1	1	48	4.4	43	20.14
12	58	1	1	48	4.4	50	29.95
13	64	1	1	48	5	36	7.76
14	11	1	1	48	5	43	24.32
15	56	1	1	48	5	50	29.90
16	71	1	1	48	5.6	36	15.12
17	65	1	1	48	5.6	43	29.18
18	21	1	1	48	5.6	50	32.49
19	69	1	1	72	4.4	36	10.82
20	20	1	1	72	4.4	43	25.94
21	53	1	1	72	4.4	50	34.46
22	39	1	1	72	5	36	11.51
23	23	1	1	72	5	43	28.86
24	44	1	1	72	5	50	34.59
25	19	1	1	72	5.6	36	17.24
26	73	1	1	72	5.6	43	34.05
27	60	1	1	72	5.6	50	35.74
28	43	1	1	24	4.4	36	2.37
29	7	1	1	24	4.4	43	7.89
30	1	1	1	24	4.4	50	23.28
31	31	1	1	24	5	36	3.07
32	41	1	1	24	5	43	10.21
33	48	1	1	24	5	50	24.36
34	77	1	1	24	5.6	36	6.78
35	54	1	1	24	5.6	43	25.41
36	6	1	1	24	5.6	50	29.63
37	55	1	1	48	4.4	36	5.60
38	61	1	1	48	4.4	43	20.16
39	50	1	1	48	4.4	50	29.99
40	78	1	1	48	5	36	7.76

41	42	1	1	48	5	43	24.40
42	45	1	1	48	5	50	30.04
43	67	1	1	48	5.6	36	15.52
44	16	1	1	48	5.6	43	28.82
45	18	1	1	48	5.6	50	32.63
46	51	1	1	72	4.4	36	10.83
47	28	1	1	72	4.4	43	26.05
48	35	1	1	72	4.4	50	34.55
49	30	1	1	72	5	36	11.61
50	24	1	1	72	5	43	28.92
51	57	1	1	72	5	50	34.66
52	36	1	1	72	5.6	36	17.17
53	76	1	1	72	5.6	43	34.06
54	62	1	1	72	5.6	50	35.84
55	47	1	1	24	4.4	36	2.39
56	38	1	1	24	4.4	43	7.92
57	10	1	1	24	4.4	50	23.07
58	3	1	1	24	5	36	3.20
59	59	1	1	24	5	43	10.19
60	70	1	1	24	5	50	24.30
61	79	1	1	24	5.6	36	6.74
62	74	1	1	24	5.6	43	25.42
63	29	1	1	24	5.6	50	29.61
64	63	1	1	48	4.4	36	5.76
65	27	1	1	48	4.4	43	20.20
66	72	1	1	48	4.4	50	29.99
67	40	1	1	48	5	36	7.76
68	81	1	1	48	5	43	24.40
69	15	1	1	48	5	50	30.22
70	12	1	1	48	5.6	36	15.26
71	66	1	1	48	5.6	43	28.83
72	52	1	1	48	5.6	50	32.67
73	8	1	1	72	4.4	36	10.79
74	25	1	1	72	4.4	43	26.02
75	13	1	1	72	4.4	50	34.45
76	68	1	1	72	5	36	11.66
77	9	1	1	72	5	43	28.91
78	26	1	1	72	5	50	34.61
79	34	1	1	72	5.6	36	17.29
80	32	1	1	72	5.6	43	34.05
81	22	1	1	72	5.6	50	35.82

Table S5. Factorial design to study the effects of reaction time, pH and temperature on the tanning reaction of B-H-D

Std Order	Run Order	Pt Type	Block	Time	pH	Temp	Colour Difference
1	46	1	1	24	4.4	36	0.61
2	4	1	1	24	4.4	43	2.12
3	33	1	1	24	4.4	50	14.00
4	75	1	1	24	5	36	0.61
5	49	1	1	24	5	43	4.60
6	80	1	1	24	5	50	16.20
7	17	1	1	24	5.6	36	1.02
8	5	1	1	24	5.6	43	6.41
9	2	1	1	24	5.6	50	22.97
10	37	1	1	48	4.4	36	1.45
11	14	1	1	48	4.4	43	7.92
12	58	1	1	48	4.4	50	18.60
13	64	1	1	48	5	36	2.64
14	11	1	1	48	5	43	8.94
15	56	1	1	48	5	50	22.79
16	71	1	1	48	5.6	36	3.53
17	65	1	1	48	5.6	43	10.00
18	21	1	1	48	5.6	50	27.76
19	69	1	1	72	4.4	36	3.26
20	20	1	1	72	4.4	43	13.87
21	53	1	1	72	4.4	50	28.72
22	39	1	1	72	5	36	3.61
23	23	1	1	72	5	43	15.23
24	44	1	1	72	5	50	30.60
25	19	1	1	72	5.6	36	5.29
26	73	1	1	72	5.6	43	16.83
27	60	1	1	72	5.6	50	32.02
28	43	1	1	24	4.4	36	0.66
29	7	1	1	24	4.4	43	2.15
30	1	1	1	24	4.4	50	14.04
31	31	1	1	24	5	36	0.71
32	41	1	1	24	5	43	4.67
33	48	1	1	24	5	50	16.20
34	77	1	1	24	5.6	36	1.04
35	54	1	1	24	5.6	43	6.43
36	6	1	1	24	5.6	50	23.00
37	55	1	1	48	4.4	36	1.54
38	61	1	1	48	4.4	43	7.93
39	50	1	1	48	4.4	50	18.61
40	78	1	1	48	5	36	2.63

41	42	1	1	48	5	43	8.94
42	45	1	1	48	5	50	22.80
43	67	1	1	48	5.6	36	3.60
44	16	1	1	48	5.6	43	10.03
45	18	1	1	48	5.6	50	27.80
46	51	1	1	72	4.4	36	3.31
47	28	1	1	72	4.4	43	13.88
48	35	1	1	72	4.4	50	29.17
49	30	1	1	72	5	36	3.67
50	24	1	1	72	5	43	15.34
51	57	1	1	72	5	50	30.69
52	36	1	1	72	5.6	36	5.34
53	76	1	1	72	5.6	43	16.89
54	62	1	1	72	5.6	50	31.90
55	47	1	1	24	4.4	36	0.71
56	38	1	1	24	4.4	43	2.12
57	10	1	1	24	4.4	50	14.05
58	3	1	1	24	5	36	0.69
59	59	1	1	24	5	43	4.61
60	70	1	1	24	5	50	16.22
61	79	1	1	24	5.6	36	1.03
62	74	1	1	24	5.6	43	6.44
63	29	1	1	24	5.6	50	22.95
64	63	1	1	48	4.4	36	1.51
65	27	1	1	48	4.4	43	7.96
66	72	1	1	48	4.4	50	18.63
67	40	1	1	48	5	36	2.67
68	81	1	1	48	5	43	8.98
69	15	1	1	48	5	50	22.42
70	12	1	1	48	5.6	36	3.65
71	66	1	1	48	5.6	43	10.01
72	52	1	1	48	5.6	50	27.80
73	8	1	1	72	4.4	36	3.38
74	25	1	1	72	4.4	43	13.91
75	13	1	1	72	4.4	50	29.44
76	68	1	1	72	5	36	3.71
77	9	1	1	72	5	43	15.32
78	26	1	1	72	5	50	30.81
79	34	1	1	72	5.6	36	5.34
80	32	1	1	72	5.6	43	16.86
81	22	1	1	72	5.6	50	31.96

Table S6. Factorial design to study the effects of reaction time, pH and temperature on the tanning reaction of L-D

Std Order	Run Order	Pt Type	Block	Time	pH	Temp	Colour Difference
1	46	1	1	24	4.4	36	2.24
2	4	1	1	24	4.4	43	7.35
3	33	1	1	24	4.4	50	13.55
4	75	1	1	24	5	36	2.53
5	49	1	1	24	5	43	12.01
6	80	1	1	24	5	50	20.16
7	17	1	1	24	5.6	36	5.77
8	5	1	1	24	5.6	43	15.09
9	2	1	1	24	5.6	50	25.23
10	37	1	1	48	4.4	36	4.89
11	14	1	1	48	4.4	43	17.06
12	58	1	1	48	4.4	50	21.33
13	64	1	1	48	5	36	5.60
14	11	1	1	48	5	43	23.45
15	56	1	1	48	5	50	27.65
16	71	1	1	48	5.6	36	10.34
17	65	1	1	48	5.6	43	28.44
18	21	1	1	48	5.6	50	33.12
19	69	1	1	72	4.4	36	7.81
20	20	1	1	72	4.4	43	22.95
21	53	1	1	72	4.4	50	28.65
22	39	1	1	72	5	36	10.97
23	23	1	1	72	5	43	26.20
24	44	1	1	72	5	50	28.98
25	19	1	1	72	5.6	36	16.51
26	73	1	1	72	5.6	43	33.73
27	60	1	1	72	5.6	50	34.19
28	43	1	1	24	4.4	36	2.24
29	7	1	1	24	4.4	43	7.35
30	1	1	1	24	4.4	50	13.48
31	31	1	1	24	5	36	2.56
32	41	1	1	24	5	43	12.08
33	48	1	1	24	5	50	20.19
34	77	1	1	24	5.6	36	5.72
35	54	1	1	24	5.6	43	15.10
36	6	1	1	24	5.6	50	25.14
37	55	1	1	48	4.4	36	4.87
38	61	1	1	48	4.4	43	17.06
39	50	1	1	48	4.4	50	21.37
40	78	1	1	48	5	36	5.59

41	42	1	1	48	5	43	23.50
42	45	1	1	48	5	50	27.59
43	67	1	1	48	5.6	36	10.49
44	16	1	1	48	5.6	43	27.36
45	18	1	1	48	5.6	50	33.08
46	51	1	1	72	4.4	36	8.17
47	28	1	1	72	4.4	43	23.00
48	35	1	1	72	4.4	50	28.70
49	30	1	1	72	5	36	10.67
50	24	1	1	72	5	43	26.20
51	57	1	1	72	5	50	28.98
52	36	1	1	72	5.6	36	16.35
53	76	1	1	72	5.6	43	33.76
54	62	1	1	72	5.6	50	34.22
55	47	1	1	24	4.4	36	2.25
56	38	1	1	24	4.4	43	7.35
57	10	1	1	24	4.4	50	13.56
58	3	1	1	24	5	36	2.54
59	59	1	1	24	5	43	12.02
60	70	1	1	24	5	50	20.21
61	79	1	1	24	5.6	36	5.68
62	74	1	1	24	5.6	43	15.15
63	29	1	1	24	5.6	50	25.27
64	63	1	1	48	4.4	36	4.86
65	27	1	1	48	4.4	43	17.06
66	72	1	1	48	4.4	50	21.41
67	40	1	1	48	5	36	5.58
68	81	1	1	48	5	43	23.57
69	15	1	1	48	5	50	27.65
70	12	1	1	48	5.6	36	10.50
71	66	1	1	48	5.6	43	28.35
72	52	1	1	48	5.6	50	33.03
73	8	1	1	72	4.4	36	8.18
74	25	1	1	72	4.4	43	23.05
75	13	1	1	72	4.4	50	28.66
76	68	1	1	72	5	36	10.47
77	9	1	1	72	5	43	26.26
78	26	1	1	72	5	50	29.09
79	34	1	1	72	5.6	36	16.54
80	32	1	1	72	5.6	43	33.75
81	22	1	1	72	5.6	50	34.24

Table S7. Factorial design to study the effects of reaction time, pH and temperature on the tanning reaction of B-L-D

Std Order	Run Order	Pt Type	Block	Time	pH	Temp	Colour Difference
1	46	1	1	24	4.4	36	8.94
2	4	1	1	24	4.4	43	13.25
3	33	1	1	24	4.4	50	15.19
4	75	1	1	24	5	36	10.11
5	49	1	1	24	5	43	15.51
6	80	1	1	24	5	50	26.28
7	17	1	1	24	5.6	36	12.22
8	5	1	1	24	5.6	43	18.25
9	2	1	1	24	5.6	50	33.25
10	37	1	1	48	4.4	36	17.61
11	14	1	1	48	4.4	43	25.88
12	58	1	1	48	4.4	50	27.68
13	64	1	1	48	5	36	18.34
14	11	1	1	48	5	43	29.38
15	56	1	1	48	5	50	33.83
16	71	1	1	48	5.6	36	20.81
17	65	1	1	48	5.6	43	32.53
18	21	1	1	48	5.6	50	34.73
19	69	1	1	72	4.4	36	18.32
20	20	1	1	72	4.4	43	28.78
21	53	1	1	72	4.4	50	31.61
22	39	1	1	72	5	36	21.88
23	23	1	1	72	5	43	32.03
24	44	1	1	72	5	50	33.43
25	19	1	1	72	5.6	36	25.23
26	73	1	1	72	5.6	43	34.28
27	60	1	1	72	5.6	50	34.20
28	43	1	1	24	4.4	36	8.99
29	7	1	1	24	4.4	43	13.25
30	1	1	1	24	4.4	50	15.15
31	31	1	1	24	5	36	10.33
32	41	1	1	24	5	43	15.45
33	48	1	1	24	5	50	26.40
34	77	1	1	24	5.6	36	12.38
35	54	1	1	24	5.6	43	18.26
36	6	1	1	24	5.6	50	32.14
37	55	1	1	48	4.4	36	17.57
38	61	1	1	48	4.4	43	25.91
39	50	1	1	48	4.4	50	28.10
40	78	1	1	48	5	36	18.48

41	42	1	1	48	5	43	29.41
42	45	1	1	48	5	50	33.25
43	67	1	1	48	5.6	36	20.89
44	16	1	1	48	5.6	43	32.45
45	18	1	1	48	5.6	50	34.79
46	51	1	1	72	4.4	36	18.33
47	28	1	1	72	4.4	43	28.87
48	35	1	1	72	4.4	50	29.67
49	30	1	1	72	5	36	21.92
50	24	1	1	72	5	43	32.02
51	57	1	1	72	5	50	33.30
52	36	1	1	72	5.6	36	25.32
53	76	1	1	72	5.6	43	34.26
54	62	1	1	72	5.6	50	34.39
55	47	1	1	24	4.4	36	8.97
56	38	1	1	24	4.4	43	13.22
57	10	1	1	24	4.4	50	15.16
58	3	1	1	24	5	36	10.27
59	59	1	1	24	5	43	15.38
60	70	1	1	24	5	50	25.76
61	79	1	1	24	5.6	36	12.18
62	74	1	1	24	5.6	43	18.27
63	29	1	1	24	5.6	50	31.97
64	63	1	1	48	4.4	36	17.62
65	27	1	1	48	4.4	43	25.87
66	72	1	1	48	4.4	50	28.18
67	40	1	1	48	5	36	18.37
68	81	1	1	48	5	43	29.38
69	15	1	1	48	5	50	31.26
70	12	1	1	48	5.6	36	20.77
71	66	1	1	48	5.6	43	32.59
72	52	1	1	48	5.6	50	34.70
73	8	1	1	72	4.4	36	18.35
74	25	1	1	72	4.4	43	29.04
75	13	1	1	72	4.4	50	29.66
76	68	1	1	72	5	36	21.80
77	9	1	1	72	5	43	32.24
78	26	1	1	72	5	50	33.45
79	34	1	1	72	5.6	36	25.38
80	32	1	1	72	5.6	43	34.31
81	22	1	1	72	5.6	50	34.56

Table S8. Preparation of AA-DHA for studying the effects of reaction time, pH and temperature on the tanning reaction

Samples	DHA (g)	Arg (g)	Boc-Arg (g)	His (g)	Boc-His (g)	Lys (g)	Boc-Lys (g)
**A-D**	0.270	0.632	—	—	—	—	—
**B-A-D**	0.540	—	0.822	—	—	—	—
**H-D**	0.810	—	—	0.629	—	—	—
**B-H-D**	0.270	—	—	—	0.765	—	—
**L-D**	0.540	—	—	—	—	0.548	—
**B-L-D**	0.810	—	—	—	—	—	0.738

Table S9. Model summary of factorial design of experiment (DOE)

AA Types	S	R-sq	R-sq(adj)	R-sq(pred)
A-D	0.0470487	100.00%	100.00%	99.99%
B-A-D	0.0765539	99.98%	99.97%	99.96%
H-D	0.0893253	100.00%	99.99%	99.99%
B-H-D	0.0908057	99.99%	99.99%	99.99%
L-D	0.138131	99.99%	99.98%	99.97%
B-L-D	0.378077	99.86%	99.79%	99.68%

Table S10. The analysis of variance (ANOVA) of A-D

Source	DF	Adj SS	Adj MS	F-Value	P-Value
Model	26	3804.94	146.344	66111.78	0.000
Linear	6	3182.97	530.496	239655.07	0.000
Time	2	525.31	262.654	118655.60	0.000
pH	2	1312.71	656.356	296513.37	0.000
Temperature	2	1344.95	672.477	303796.23	0.000
2-Way Interaction	12	584.13	48.678	21990.42	0.000
Time*pH	4	60.47	15.117	6829.36	0.000
Time*Temp	4	121.49	30.372	13720.63	0.000
pH*Temp	4	402.17	100.544	45421.28	0.000
3-Way interaction	8	37.83	4.729	2136.35	0.000
Time*pH*Temp	8	37.83	4.729	2136.35	0.000
Error	54	0.12	0.002		
Total	80	3805.06			

Table S11. The analysis of variance (ANOVA) of B-A-D

Source	DF	Adj SS	Adj MS	F-Value	P-Value
Model	26	8136.85	312.96	16402.09	0.000
Linear	6	7795.93	1299.32	68097.77	0.000
Time	2	1925.35	962.67	50453.99	0.000
pH	2	970.01	485.01	25419.26	0.000
Temperature	2	4900.57	2450.29	128420.07	0.000
2-Way Interaction	12	272.82	22.74	1191.57	0.000
Time*pH	4	22.98	5.74	301.09	0.000
Time*Temp	4	195.41	48.85	2560.32	0.000
pH*Temp	4	54.44	13.61	713.28	0.000
3-Way interaction	8	68.09	8.51	446.11	0.000
Time*pH*Temp	8	68.09	8.51	446.11	0.000
Error	54	1.03	0.02		
Total	80	8137.88			

Table S12. The analysis of variance (ANOVA) of H-D

Source	DF	Adj SS	Adj MS	F-Value	P-Value
Model	26	9401.27	361.59	45317.29	0.000
Linear	6	8954.80	1492.47	187049.12	0.000
Time	2	1719.04	859.52	107722.48	0.000
pH	2	776.97	388.49	48688.45	0.000
Temperature	2	6458.79	3229.40	404736.44	0.000
2-Way Interaction	12	324.31	27.03	3387.07	0.000
Time*pH	4	53.97	13.49	1690.86	0.000
Time*Temp	4	118.26	29.57	3705.48	0.000
pH*Temp	4	152.08	38.02	4764.87	0.000
3-Way interaction	8	122.16	15.27	1913.74	0.000
Time*pH*Temp	8	122.16	15.27	1913.74	0.000
Error	54	0.43	0.01		
Total	80	9401.70			

Table S13. The analysis of variance (ANOVA) of B-H-D

Source	DF	Adj SS	Adj MS	F-Value	P-Value
Model	26	8026.88	308.73	37440.96	0.000
Linear	6	7664.79	1277.46	154925.32	0.000
Time	2	1110.61	555.30	67344.79	0.000
pH	2	203.84	101.92	12360.42	0.000
Temperature	2	6350.34	3175.17	385070.77	0.000
2-Way Interaction	12	321.51	26.79	3249.29	0.000
Time*pH	4	11.81	2.95	357.92	0.000
Time*Temp	4	234.96	58.74	7123.71	0.000
pH*Temp	4	74.75	18.69	2266.24	0.000
3-Way interaction	8	40.58	5.07	615.20	0.000
Time*pH*Temp	8	40.58	5.07	615.20	0.000
Error	54	0.45	0.01		
Total	80	8027.32			

Table S14. The analysis of variance (ANOVA) of L-D

Source	DF	Adj SS	Adj MS	F-Value	P-Value
Model	26	8136.85	312.96	16402.09	0.000
Linear	6	7795.93	1299.32	68097.77	0.000
Time	2	1925.35	962.67	50453.99	0.000
pH	2	970.01	485.01	25419.26	0.000
Temperature	2	4900.57	2450.29	128420.07	0.000
2-Way Interaction	12	272.82	22.74	1191.57	0.000
Time*pH	4	22.98	5.74	301.09	0.000
Time*Temp	4	195.41	48.85	2560.32	0.000
pH*Temp	4	54.44	13.61	713.28	0.000
3-Way interaction	8	68.09	8.51	446.11	0.000
Time*pH*Temp	8	68.09	8.51	446.11	0.000
Error	54	1.03	0.02		
Total	80	8137.88			

Table S15. The analysis of variance (ANOVA) of B-L-D

Source	DF	Adj SS	Adj MS	F-Value	P-Value
Model	26	5373.24	206.66	1445.78	0.000
Linear	6	4959.70	826.62	5782.88	0.000
Time	2	2158.88	1079.44	7551.59	0.000
pH	2	573.76	286.88	2006.98	0.000
Temperature	2	2227.06	1113.53	7790.08	0.000
2-Way Interaction	12	273.21	22.77	159.28	0.000
Time*pH	4	28.34	7.09	49.57	0.000
Time*Temp	4	175.42	43.85	306.80	0.000
pH*Temp	4	69.45	17.36	121.46	0.000
3-Way interaction	8	140.33	17.54	122.71	0.000
Time*pH*Temp	8	140.33	17.54	122.71	0.000
Error	54	7.72	0.14		
Total	80	5380.96			

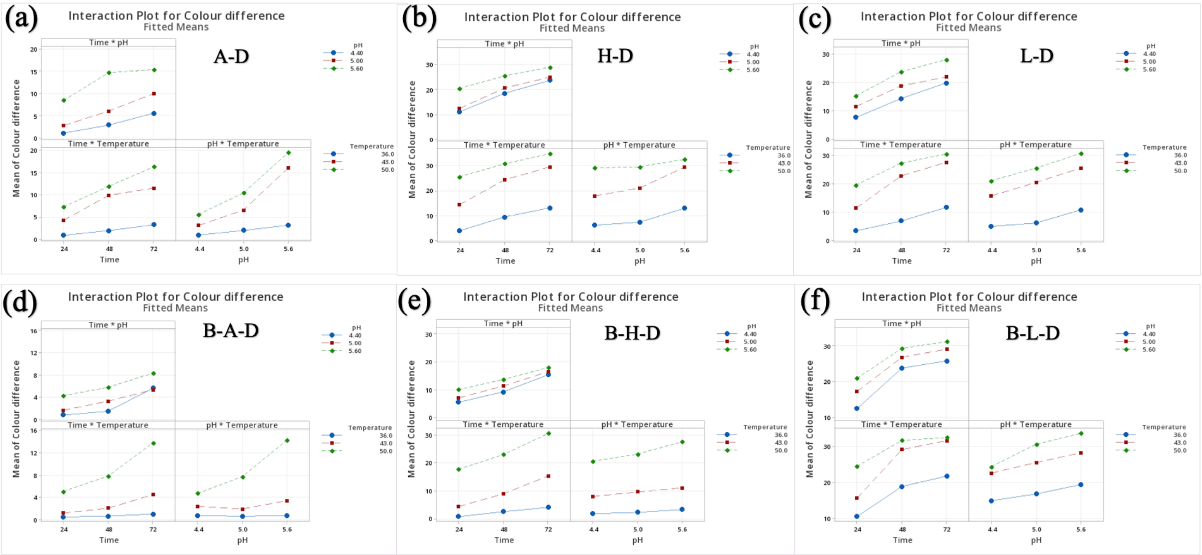

Figure S1. Interaction plots of reaction time, pH and temperature on the colour difference of (a) A-D (b) H-D (c) L-D (d) B-A-D (e) B-H-D and (f) B-L-D

Table S16. CIELAB results of six model systems obtained at pH 4.4 and 36 °C

AA Types	Time (h)	Colour Difference	Std Dev	*a**	Std Dev	*b**	Std Dev
A-D	24	0.69	0.04	0.77	0.03	-4.55	0.04
B-A-D	24	0.38	0.02	0.77	0.02	-4.18	0.03
H-D	24	2.39	0.08	0.10	0.04	-1.80	0.01
B-H-D	24	0.66	0.05	0.55	0.10	-3.96	0.03
L-D	24	2.24	0.01	0.13	0.01	-2.15	0.06
B-L-D	24	8.97	0.05	-0.44	0.01	4.67	0.02
A-D	48	0.77	0.05	0.71	0.02	-4.32	0.35
B-A-D	48	0.49	0.03	0.82	0.02	-4.46	0.01
H-D	48	5.68	0.08	-0.63	0.01	1.41	0.08
B-H-D	48	1.5	0.04	0.20	0.01	-2.77	0.03
L-D	48	4.87	0.02	-0.31	0.01	0.67	0.02
B-L-D	48	17.6	0.06	0.01	0.00	13.17	0.04
A-D	72	1.54	0.05	0.55	0.01	-3.93	0.16
B-A-D	72	0.73	0.04	0.77	0.02	-4.69	0.09
H-D	72	10.81	0.08	-1.23	0.01	6.46	0.07
B-H-D	72	3.32	0.05	-0.13	0.01	-0.91	0.13
L-D	72	8.05	0.21	-0.79	0.04	4.00	0.26
B-L-D	72	18.33	0.06	-0.14	0.01	13.80	0.05

Table S17. CIELAB results of six model systems obtained at pH 5.0 and 36 °C

AA Types	Time (h)	Colour Difference	Std Dev	*a**	Std Dev	*b**	Std Dev
A-D	24	0.94	0.18	0.85	0.03	-4.85	0.11
B-A-D	24	0.49	0.00	0.84	0.02	-4.51	0.02
H-D	24	3.11	0.02	-0.18	0.01	-1.19	0.02
B-H-D	24	0.67	0.06	0.68	0.04	-4.15	0.11
L-D	24	2.54	0.01	0.14	0.01	-1.63	0.01
B-L-D	24	10.23	0.03	-0.50	0.02	6.01	0.02
A-D	48	1.81	0.01	0.53	0.01	-3.37	0.01
B-A-D	48	0.73	0.02	0.83	0.01	-4.68	0.00
H-D	48	7.76	0.00	-0.93	0.02	3.47	0.02
B-H-D	48	2.65	0.02	0.00	0.01	-1.56	0.02
L-D	48	5.59	0.01	-0.40	0.01	1.32	0.01
B-L-D	48	18.40	0.01	-0.16	0.01	13.84	0.01
A-D	72	3.29	0.02	0.59	0.02	-3.44	0.51
B-A-D	72	0.78	0.10	0.84	0.02	-4.62	0.05
H-D	72	11.59	0.02	-1.49	0.01	7.16	0.02
B-H-D	72	3.66	0.06	-0.48	0.03	-0.73	0.07
L-D	72	10.70	0.21	1.15	0.01	6.61	0.21
B-L-D	72	21.87	0.03	0.01	0.01	17.31	0.02

Table S18. CIELAB results of six model systems obtained at pH 5.6 and 36 °C

AA Types	Time (h)	Colour Difference	Std Dev	*a**	Std Dev	*b**	Std Dev
A-D	24	1.14	0.01	0.83	0.07	-4.90	0.45
B-A-D	24	0.71	0.10	0.58	0.00	-3.78	0.08
H-D	24	6.69	0.13	-0.80	0.01	2.39	0.12
B-H-D	24	1.03	0.01	0.43	0.01	-3.65	0.02
L-D	24	5.72	0.05	-0.44	0.00	1.51	0.04
B-L-D	24	12.26	0.11	-0.57	0.01	7.94	0.09
A-D	48	3.46	0.02	-0.03	0.01	-0.74	0.02
B-A-D	48	0.86	0.10	0.82	0.01	-4.79	0.09
H-D	48	15.30	0.20	-1.47	0.01	10.90	0.18
B-H-D	48	3.59	0.06	-0.24	0.01	-0.64	0.07
L-D	48	10.44	0.09	-1.00	0.01	6.15	0.09
B-L-D	48	20.82	0.07	0.31	0.01	16.29	0.07
A-D	72	5.05	0.04	-0.49	0.01	0.81	0.04
B-A-D	72	0.90	0.09	0.71	0.01	-4.58	0.04
H-D	72	17.23	0.06	-1.36	0.01	12.80	0.05
B-H-D	72	5.33	0.03	-0.60	0.01	1.03	0.03
L-D	72	16.47	0.10	-1.30	0.01	12.10	0.09
B-L-D	72	25.31	0.07	1.41	0.01	20.31	0.08

Table S19. CIELAB results of six model systems obtained at pH 4.4 and 43 °C

AA Types	Time (h)	Colour Difference	Std Dev	*a**	Std Dev	*b**	Std Dev
A-D	24	0.79	0.02	0.58	0.02	-3.34	0.03
B-A-D	24	0.48	0.02	0.84	0.02	-4.48	0.03
H-D	24	7.90	0.02	-0.95	0.01	3.54	0.02
B-H-D	24	2.13	0.02	0.15	0.02	-2.06	0.02
L-D	24	7.35	0.00	-0.49	0.02	3.08	0.01
B-L-D	24	13.24	0.02	-0.35	0.00	8.71	0.02
A-D	48	3.00	0.01	0.08	0.01	-1.19	0.01
B-A-D	48	1.30	0.03	0.58	0.01	-3.15	0.01
H-D	48	20.17	0.03	-1.38	0.02	15.66	0.02
B-H-D	48	7.93	0.02	-0.80	0.02	3.58	0.02
L-D	48	17.06	0.00	-0.79	0.01	12.60	0.02
B-L-D	48	25.88	0.02	1.61	0.01	20.86	0.02
A-D	72	5.65	0.05	-0.49	0.02	1.29	0.05
B-A-D	72	2.23	0.01	0.19	0.01	-1.96	0.01
H-D	72	26.00	0.05	-0.30	0.02	21.38	0.04
B-H-D	72	13.89	0.02	-1.38	0.02	9.46	0.02
L-D	72	23.00	0.05	-0.10	0.03	18.51	0.04
B-L-D	72	28.90	0.13	2.51	0.02	23.73	0.21

Table S20. CIELAB results of six model systems obtained at pH 5.0 and 43 °C

AA Types	Time (h)	Colour Difference	Std Dev	*a**	Std Dev	*b**	Std Dev
A-D	24	2.42	0.01	0.43	0.01	-2.59	0.03
B-A-D	24	1.45	0.20	0.76	0.02	-4.47	0.10
H-D	24	10.16	0.07	-1.19	0.02	5.84	0.09
B-H-D	24	4.63	0.04	-0.30	0.01	0.33	0.05
L-D	24	12.03	0.04	-0.88	0.01	7.86	0.04
B-L-D	24	15.45	0.07	-0.30	0.02	11.27	0.06
A-D	48	7.59	0.01	-0.67	0.01	3.28	0.01
B-A-D	48	1.68	0.01	0.41	0.01	-2.45	0.01
H-D	48	24.38	0.04	-0.71	0.02	19.90	0.04
B-H-D	48	8.96	0.03	-0.92	0.01	4.66	0.02
L-D	48	23.51	0.06	-0.11	0.03	19.05	0.05
B-L-D	48	29.39	0.02	3.08	0.01	24.00	0.02
A-D	72	9.83	0.02	-1.06	0.02	5.52	0.02
B-A-D	72	2.77	0.01	0.10	0.01	-1.40	0.01
H-D	72	28.90	0.03	1.09	0.02	24.22	0.06
B-H-D	72	15.30	0.06	-1.38	0.02	10.90	0.05
L-D	72	26.22	0.04	0.69	0.01	21.55	0.03
B-L-D	72	32.10	0.12	4.62	0.02	26.50	0.19

Table S21. CIELAB results of six model systems obtained at pH 5.6 and 43 °C

AA Types	Time (h)	Colour Difference	Std Dev	*a**	Std Dev	*b**	Std Dev
A-D	24	9.73	0.01	-0.98	0.01	5.41	0.00
B-A-D	24	1.80	0.03	0.78	0.08	-4.47	0.23
H-D	24	25.43	0.03	-0.57	0.01	20.96	0.02
B-H-D	24	6.43	0.02	-0.71	0.03	2.13	0.01
L-D	24	15.11	0.06	0.96	0.01	12.76	0.04
B-L-D	24	18.26	0.01	-0.12	0.01	13.65	0.01
A-D	48	19.21	0.01	-1.06	0.01	14.63	0.01
B-A-D	48	3.37	0.01	-0.01	0.02	-0.90	0.01
H-D	48	28.94	0.09	2.78	0.02	24.01	0.09
B-H-D	48	10.01	0.02	-1.07	0.02	5.68	0.02
L-D	48	28.05	0.05	2.78	0.05	22.72	0.04
B-L-D	48	32.53	0.07	6.07	0.02	26.06	0.06
A-D	72	20.35	0.03	-1.12	0.03	14.62	0.03
B-A-D	72	5.17	0.02	-0.50	0.02	0.91	0.01
H-D	72	34.05	0.05	3.84	0.01	29.05	0.01
B-H-D	72	16.86	0.03	-1.47	0.01	12.37	0.02
L-D	72	33.75	0.03	7.63	0.02	26.95	0.02
B-L-D	72	34.33	0.06	7.51	0.13	27.07	0.33

Table S22. CIELAB results of six model systems obtained at pH 4.4 and 50°C

AA Types	Time (h)	Colour Difference	Std Dev	*a**	Std Dev	*b**	Std Dev
A-D	24	2.08	0.06	0.25	0.02	-2.39	0.07
B-A-D	24	1.67	0.09	0.61	0.01	-3.69	0.06
H-D	24	23.25	0.08	-0.82	0.02	18.90	0.08
B-H-D	24	14.03	0.03	-1.23	0.02	9.80	0.05
L-D	24	13.53	0.04	-0.81	0.00	9.34	0.04
B-L-D	24	15.17	0.34	-0.36	0.01	10.71	0.36
A-D	48	5.14	0.00	-0.54	0.00	0.80	0.01
B-A-D	48	2.84	0.01	0.07	0.01	-1.33	0.01
H-D	48	29.98	0.02	-1.70	0.01	25.46	0.02
B-H-D	48	18.61	0.02	-1.26	0.01	14.28	0.02
L-D	48	21.37	0.04	-0.24	0.01	16.97	0.06
B-L-D	48	27.98	0.27	2.55	0.05	22.95	0.28
A-D	72	9.78	0.01	-1.40	0.01	5.42	0.01
B-A-D	72	7.92	0.02	-0.96	0.01	3.64	0.02
H-D	72	34.48	0.06	0.35	0.01	29.81	0.06
B-H-D	72	29.11	0.10	0.34	0.04	24.71	0.11
L-D	72	28.67	0.03	2.14	0.02	23.44	0.15
B-L-D	72	30.32	1.12	6.21	0.48	23.18	1.18

Table S23. CIELAB results of six model systems obtained at pH 5.0 and 50 °C

AA Types	Time (h)	Colour Difference	Std Dev	*a**	Std Dev	*b**	Std Dev
A-D	24	5.23	0.02	-0.25	0.01	1.01	0.02
B-A-D	24	3.16	0.01	0.13	0.02	0.98	0.01
H-D	24	24.33	0.03	-1.02	0.02	20.08	0.03
B-H-D	24	16.21	0.01	-1.40	0.01	11.89	0.01
L-D	24	20.19	0.02	-0.36	0.01	15.93	0.02
B-L-D	24	26.15	0.59	2.19	0.30	21.52	0.81
A-D	48	9.03	0.01	4.72	0.01	4.81	0.01
B-A-D	48	7.59	0.02	-0.84	0.02	3.26	0.01
H-D	48	30.05	0.16	1.00	0.03	25.33	0.19
B-H-D	48	22.61	0.02	-0.38	0.02	18.09	0.02
L-D	48	27.63	0.03	1.67	0.01	22.78	0.03
B-L-D	48	32.78	1.35	11.67	1.33	21.35	1.21
A-D	72	17.05	0.02	-1.54	0.03	12.63	0.01
B-A-D	72	12.60	0.04	-1.27	0.61	8.23	0.09
H-D	72	34.62	0.04	4.92	0.03	29.00	0.04
B-H-D	72	30.70	0.06	1.25	0.01	25.99	0.05
L-D	72	29.02	0.07	3.67	0.03	23.72	0.06
B-L-D	72	33.39	0.08	12.84	0.01	22.03	0.06

Table S24. CIELAB results of six model systems obtained at pH 5.6 and 50°C

AA Types	Time (h)	Colour Difference	Std Dev	*a**	Std Dev	*b**	Std Dev
A-D	24	14.74	0.13	1.08	0.01	5.09	0.03
B-A-D	24	10.55	0.01	-1.31	0.01	6.20	0.02
H-D	24	29.57	0.17	-2.46	0.03	25.23	0.16
B-H-D	24	22.98	0.02	-1.09	0.00	18.58	0.03
L-D	24	25.21	0.07	1.66	0.02	20.37	0.07
B-L-D	24	32.45	0.02	6.46	0.01	26.03	0.01
A-D	48	21.68	0.04	-0.87	0.02	17.20	0.04
B-A-D	48	13.18	0.02	-1.50	0.02	8.67	0.04
H-D	48	32.60	0.21	2.85	0.03	27.48	0.24
B-H-D	48	27.78	0.21	-0.38	0.04	23.26	0.18
L-D	48	33.08	0.04	10.50	0.01	23.43	0.03
B-L-D	48	34.74	0.60	12.28	0.14	24.76	0.76
A-D	72	22.15	0.02	-1.17	0.01	17.60	0.01
B-A-D	72	19.07	0.29	-1.94	0.00	14.73	0.29
H-D	72	35.80	0.01	10.95	0.01	27.15	0.01
B-H-D	72	31.96	0.36	1.55	0.11	27.13	0.35
L-D	72	34.17	0.02	13.00	0.01	22.49	0.02
B-L-D	72	35.22	0.10	8.88	0.11	26.70	0.11

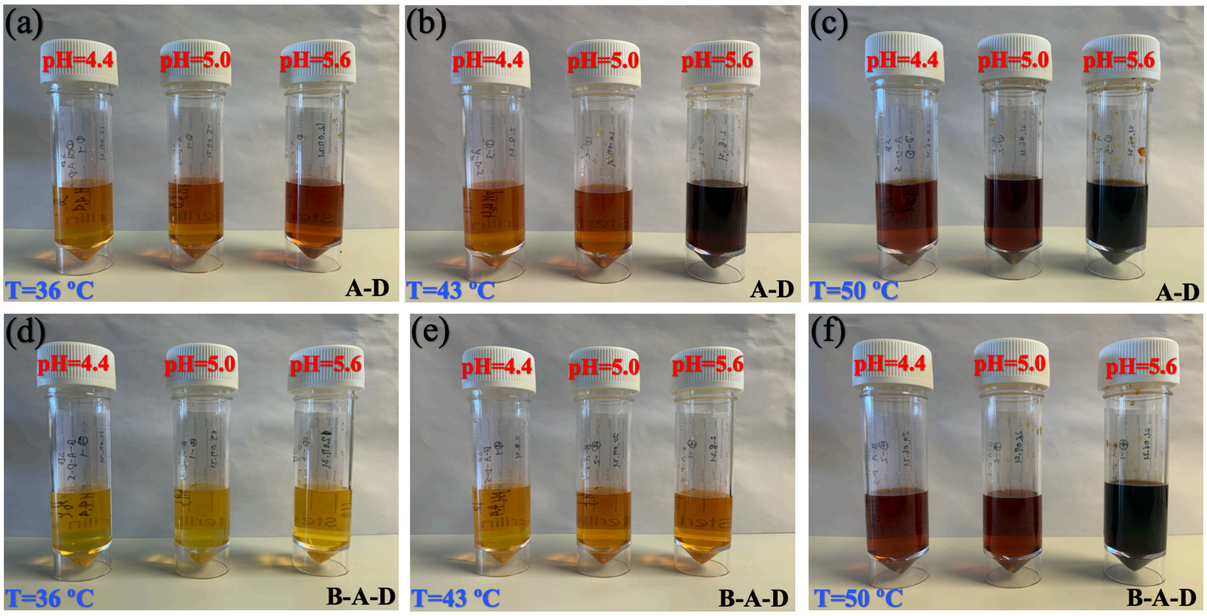

Figure S2. Sample images of A-D and B-A-D at 72h with varying pH and temperature: (a) A-D, 36 °C (b) A-D, 43°C (c) A-D, 50°C (d) B-A-D, 36°C (e) B-A-D, 43°C and (f) B-A-D, 50 °C

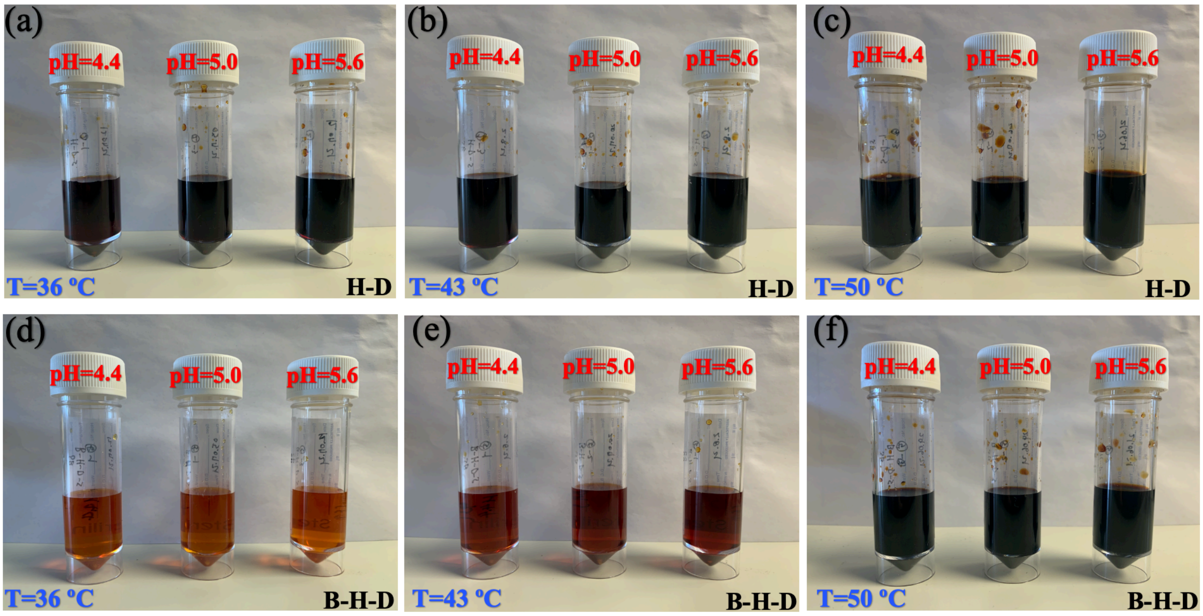

Figure S3. Sample images of H-D and B-H-D at 72h with varying pH and temperature: (a) H-D, 36 °C (b) H-D, 43°C (c) H-D, 50°C (d) B-H-D, 36°C (e) B-H-D, 43°C and (f) B-H-D, 50 °C

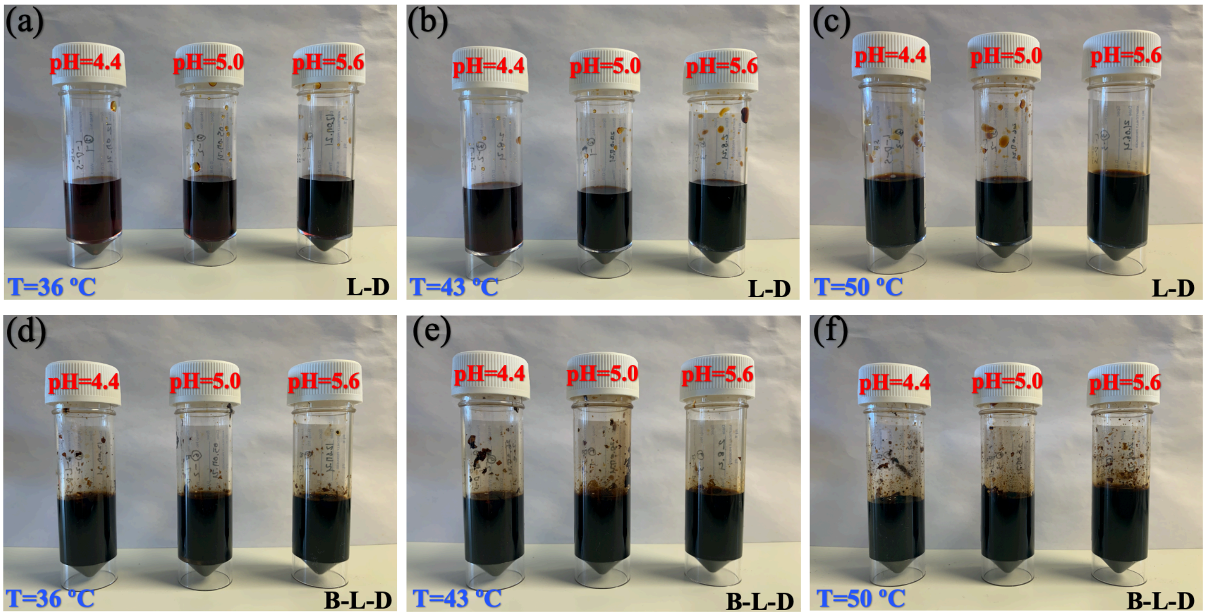

Figure S4. Sample images of L-D and B-L-D at 72h with varying pH and temperature: (a) L-D, 36 °C (b) L-D, 43°C (c) L-D, 50°C (d) B-L-D, 36°C (e) B-L-D, 43°C and (f) B-L-D, 50 °C

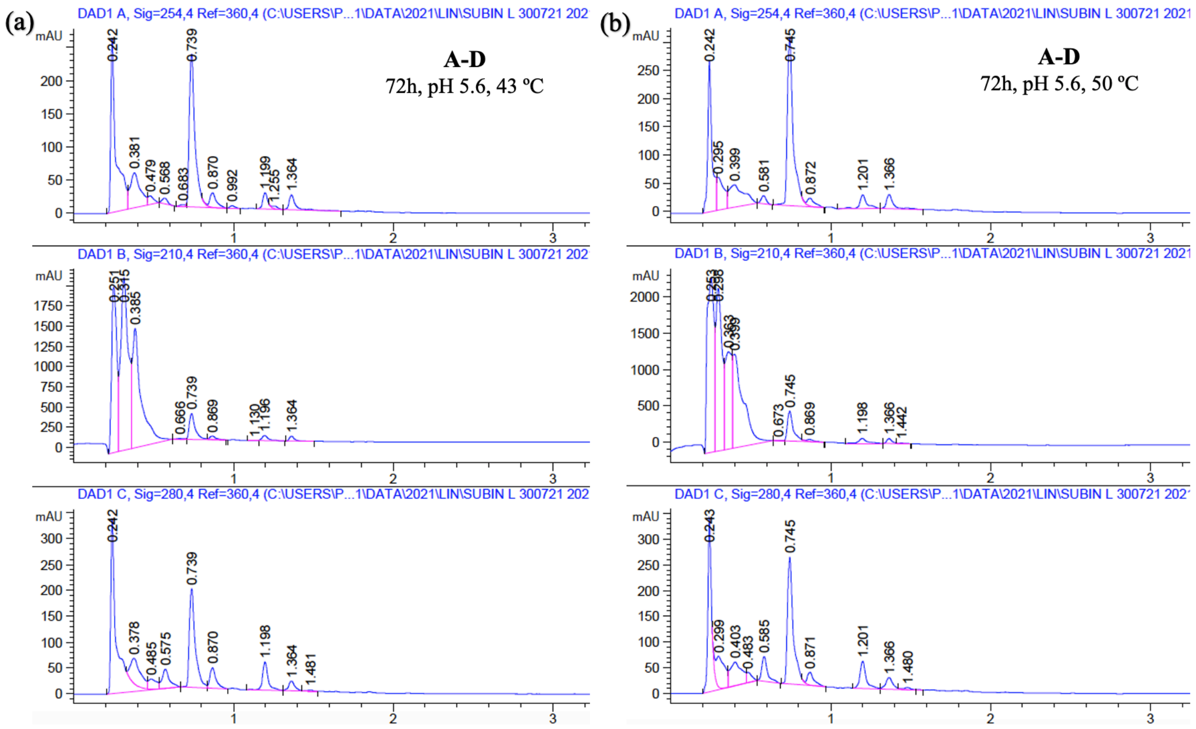

Figure S5. Analytical HPLC of A-D at (a) 72 h, pH 5.6, 43 °C and (b) 72 h, pH 5.6, 50 °C (1 µL, rapid 5-50% MeCN, 5 min, signals at 254, 210 and 280 nm)

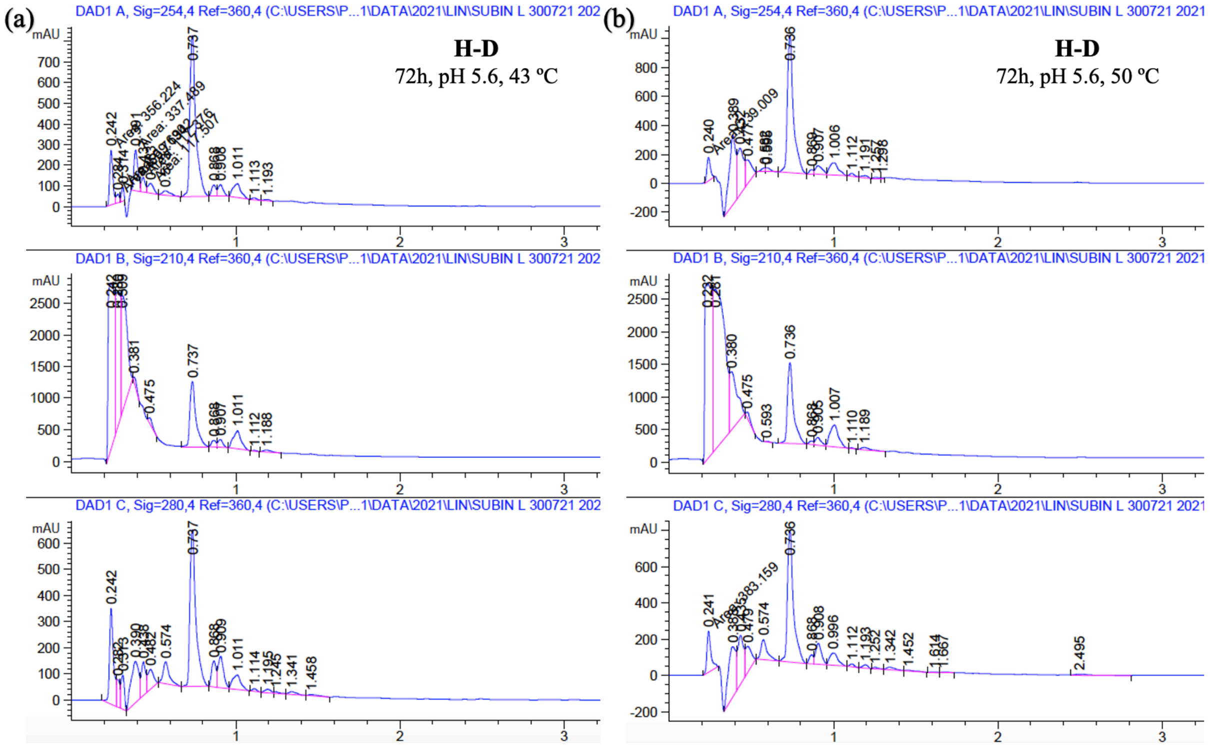

Figure S6. Analytical HPLC of H-D at (a) 72 h, pH 5.6, 43 °C and (b) 72 h, pH 5.6, 50 °C (1 µL, rapid 5-50% MeCN, 5 min, signals at 254, 210 and 280 nm)

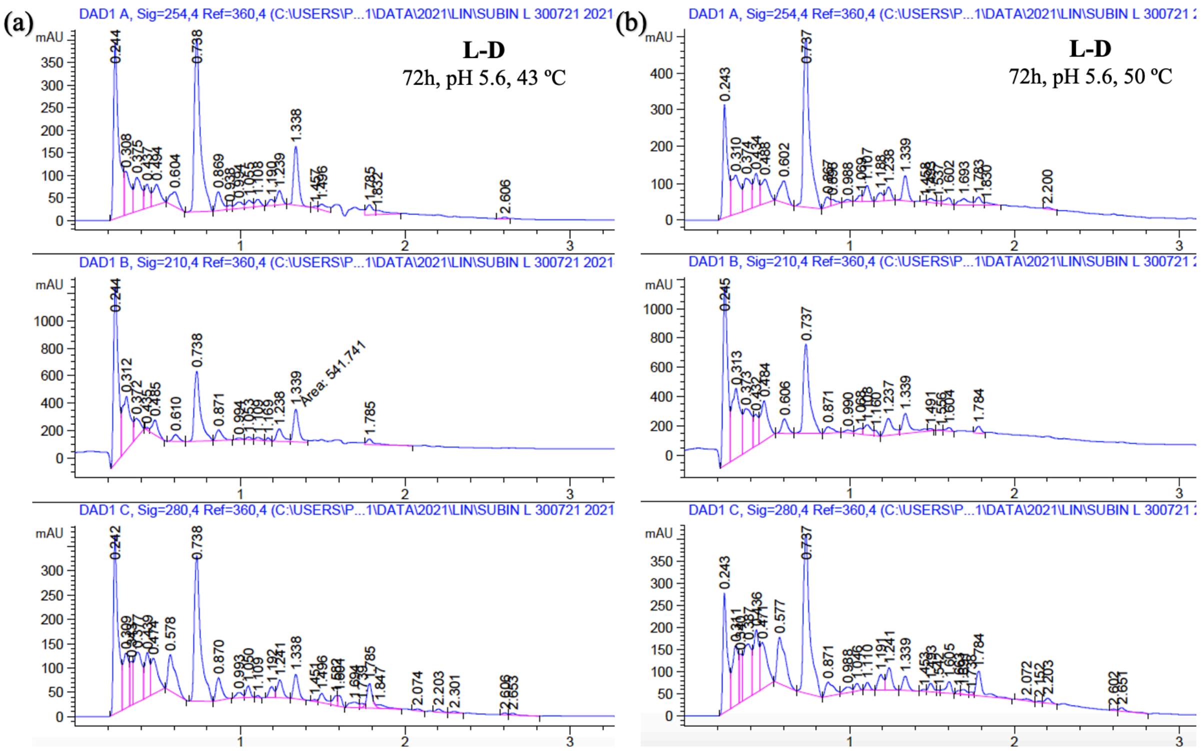

Figure S7. Analytical HPLC of L-D at (a) 72 h, pH 5.6, 43 °C and (b) 72 h, pH 5.6, 50 °C (1 μL, rapid 5-50% MeCN, 5 min, signals at 254, 210 and 280 nm)

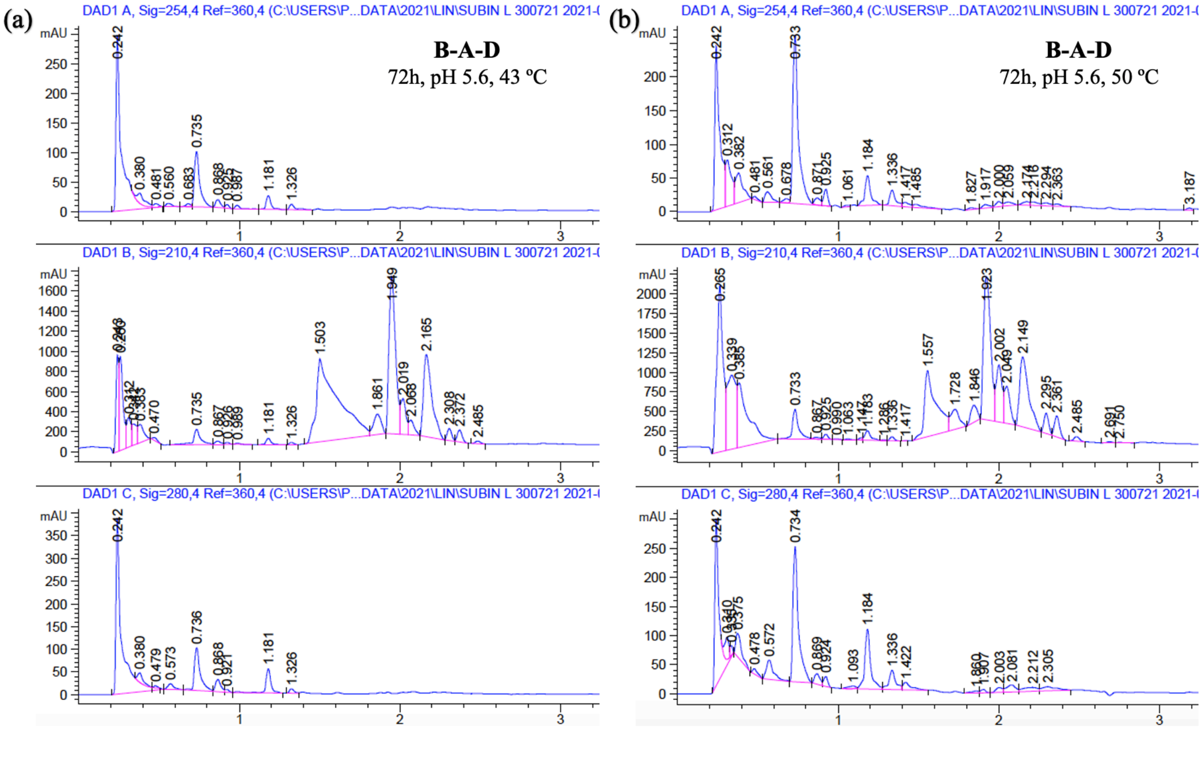

Figure S8. Analytical HPLC of B-A-D at (a) 72 h, pH 5.6, 43 °C and (b) 72 h, pH 5.6, 50 °C (1 μL, rapid 5-50% MeCN, 5 min, signals at 254, 210 and 280 nm)

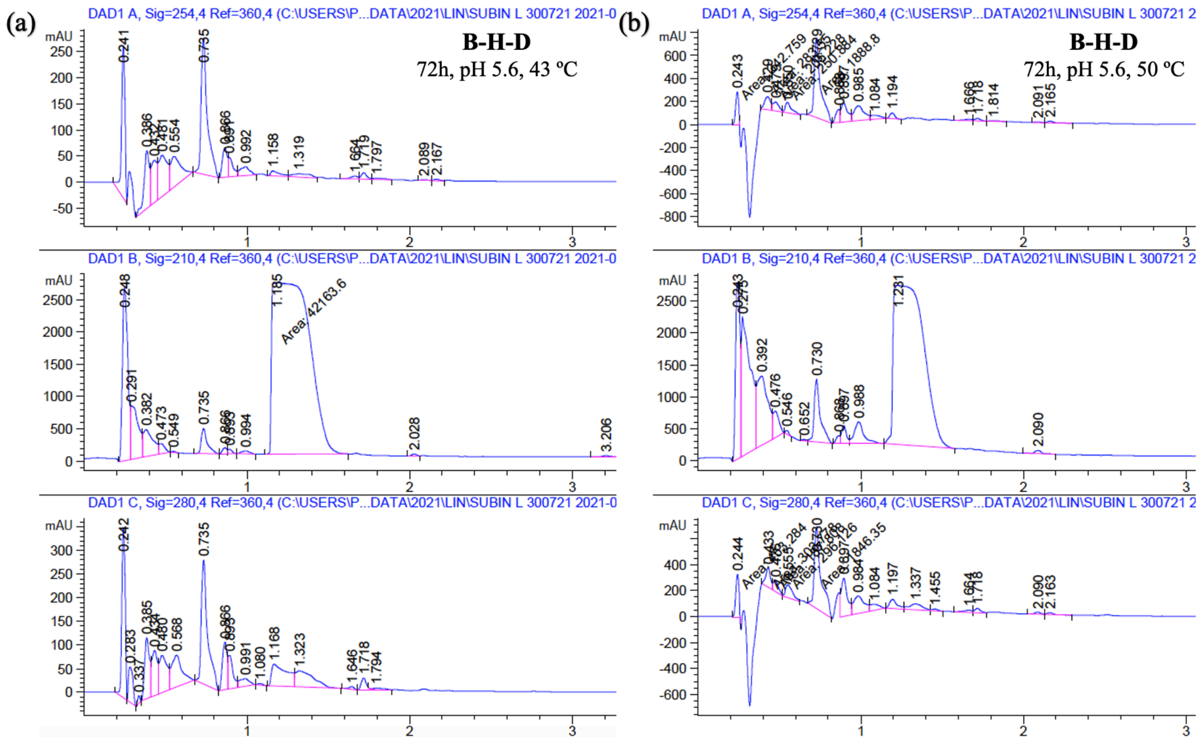

Figure S9. Analytical HPLC of B-H-D at (a) 72 h, pH 5.6, 43 °C and (b) 72 h, pH 5.6, 50 °C (1 µL, rapid 5-50% MeCN, 5 min, signals at 254, 210 and 280 nm)

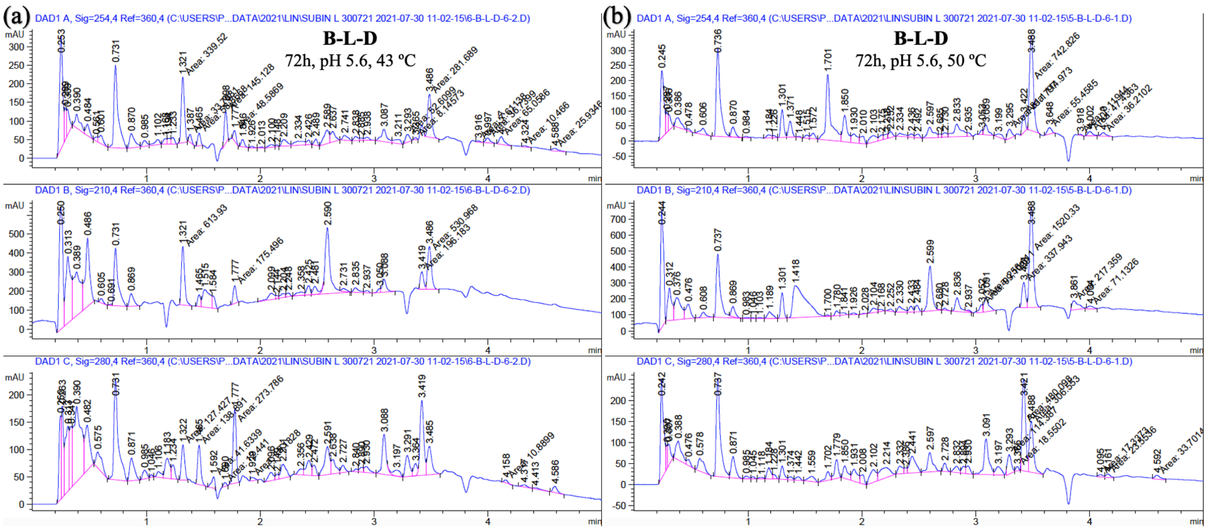

Figure S10. Analytical HPLC of B-L-D at (a) 72 h, pH 5.6, 43 °C and (b) 72 h, pH 5.6, 50 °C (1 µL, rapid 5-50% MeCN, 5 min, signals at 254, 210 and 280 nm)

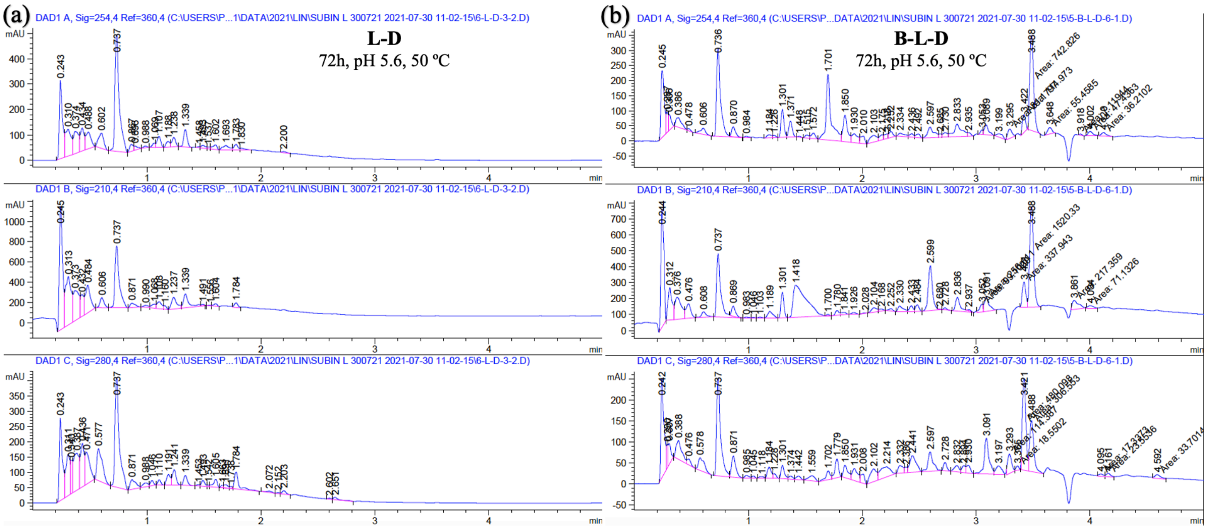

Figure S11. Analytical HPLC of (a) L-D at 72 h, pH 5.6, 50 °C and (b) B-L-D at 72 h, pH 5.6, 50 °C (1 μL, rapid 5-50% MeCN, 5 min, signals at 254, 210 and 280 nm)
